# Recognition of Melanocytes in Immuno-Neuroendocrinology and Circadian Rhythms: Beyond the Conventional Melanin Synthesis

**DOI:** 10.3390/cells11132082

**Published:** 2022-06-30

**Authors:** Yan-Yan Chen, Li-Ping Liu, Hang Zhou, Yun-Wen Zheng, Yu-Mei Li

**Affiliations:** 1 Institute of Regenerative Medicine, Affiliated Hospital of Jiangsu University, Jiangsu University, Zhenjiang 212001, China; chenyanyan0525@stmail.ujs.edu.cn (Y.-Y.C.); liuliping@ujs.edu.cn (L.-P.L.); zhouhang@stmail.ujs.edu.cn (H.Z.); 2 Department of Dermatology, Affiliated Hospital of Jiangsu University, Jiangsu University, Zhenjiang 212001, China; 3 Guangdong Provincial Key Laboratory of Large Animal Models for Biomedicine, School of Biotechnology and Health Sciences, Wuyi University, Jiangmen 529020, China; 4 Department of Medicinal and Life Sciences, Faculty of Pharmaceutical Sciences, Tokyo University of Science, Noda 278-8510, Japan; 5 School of Medicine, Yokohama City University, Yokohama 234-0006, Japan; 6 Faculty of Medicine, University of Tsukuba, Tsukuba 305-8575, Japan; 7 Center for Stem Cell Biology and Regenerative Medicine, Institute of Medical Science, The University of Tokyo, Tokyo 108-8639, Japan

**Keywords:** melanocytes, neuroendocrinology, circadian rhythm, photoentrainment, homeostasis, regulatory network, sensory functions, opsins, extracutaneous pigment cell, innate immunity

## Abstract

Melanocytes produce melanin to protect the skin from UV-B radiation. Notwithstanding, the spectrum of their functions extends far beyond their well-known role as melanin production factories. Melanocytes have been considered as sensory and computational cells. The neurotransmitters, neuropeptides, and other hormones produced by melanocytes make them part of the skin’s well-orchestrated and complex neuroendocrine network, counteracting environmental stressors. Melanocytes can also actively mediate the epidermal immune response. Melanocytes are equipped with ectopic sensory systems similar to the eye and nose and can sense light and odor. The ubiquitous inner circadian rhythm controls the body’s basic physiological processes. Light not only affects skin photoaging, but also regulates inner circadian rhythms and communicates with the local neuroendocrine system. Do melanocytes “see” light and play a unique role in photoentrainment of the local circadian clock system? Why, then, are melanocytes responsible for so many mysterious functions? Do these complex functional devices work to maintain homeostasis locally and throughout the body? In addition, melanocytes have also been shown to be localized in internal sites such as the inner ear, brain, and heart, locations not stimulated by sunlight. Thus, what can the observation of extracutaneous melanocytes tell us about the “secret identity” of melanocytes? While the answers to some of these intriguing questions remain to be discovered, here we summarize and weave a thread around available data to explore the established and potential roles of melanocytes in the biological communication of skin and systemic homeostasis, and elaborate on important open issues and propose ways forward.

## 1. Introduction

Melanocytes can be defined as neuroectodermic dendritic cells with unique melanin synthesis capabilities. They reside in the basal cell layer, the outermost layer of the skin, accounting for nearly 2–4% of epidermal cells and 8–10% of basal layer cells in the epidermis [1,2]. Melanocytes are well known for synthesizing the melanin responsible for camouflage, sun and radiation protection, harvesting energy, scavenging metal ions and free radicals [3]. In addition, melanin manifests special properties and functions in general health, including antioxidant, anti-inflammatory, immune regulation, hepatic, gastrointestinal, and hypoglycemic benefits [3,4]. Therefore, melanin is an interesting but undervalued multifunctional molecule that accomplishes far more than coloration and protection from ultraviolet B (UV-B) radiation. What, then, is the nature of the cells that generate melanin?

Melanocytes not only exert their action via synthesizing melanin but have been demonstrated to have other non-classical physiological functions that are less well known, whether considered from an evolutionary perspective, their strategic position in the epidermis, or a close relationship between stress and skin diseases [5,6,7]. In addition, melanocytes are also located in the inner ears, heart, brain, and other unexposed organs, and this distribution has also indicated that melanocytes exert some non-pigmented functions [8]. Therefore, this review summarizes the non-classical functions of skin melanocytes, including neuroendocrine, immune regulation, and local perception of light and odor, as well as the functions of melanocytes located outside the skin. Considering that its embryologic ectoderm-origin sibling is the brain, the complexity of the melanocyte is not surprising [5,9]. We further call for attention to research in this direction as an instructive model for exploring and therapeutically manipulating the comprehensive regulation networks of melanocytes in the skin physiology or even throughout the body.

## 2. Melanocytes and the Endocrine System

Stress has been anecdotally associated with skin conditions and diseases [10]. It is generally believed that stress can challenge skin homeostasis, worsen skin conditions including impaired skin barrier function, hair greying, compromised wound healing, and even trigger or aggravate various diseases such as acne, atopic dermatitis, psoriasis, alopecia areata, and vitiligo [11,12,13,14,15]. Accumulating evidence has shown that besides being potential targets of stress, melanocytes have evolved a precisely coordinated neuroendocrine system to actively cope with a variety of stress situations [6,16,17]. Over two decades ago, it was proposed that normal epidermal melanocytes have the sensory and regulatory ability to maintain cutaneous homeostasis [7]. While this concept is still under development, it has been widely acknowledged that melanocytes are endowed with regulation and adaptation mechanisms, particularly for their neuroendocrine function, including secreting a considerably wide variety of classic hormones and neurotransmitters and expressing the corresponding receptors [9,18].

### 2.1. Hypothalamic-Pituitary-Adrenal (HPA) Axis Homolog

The hypothalamic-pituitary-adrenal (HPA) axis, an essential component of the neuroendocrine system, consists of a complex and robust negative feedback loop to control the body’s systemic stress response [10]. Recently, numerous studies have revealed that there is a functional homolog of the HPA axis in cutaneous melanocytes that can produce classic hormones, including corticotropin-releasing hormone (CRH), proopiomelanocortin (POMC) [19], adrenocorticotropic hormone (ACTH), β-endorphins, α-melanocyte-stimulating hormone (α-MSH), and their corresponding receptors, such as CRH receptor (CRH-R), the MSH receptor (MC1-R) [20,21,22,23,24,25]. Using the immunohistochemical and in-situ RT-PCR methods, these peptides in the skin were further proved to be produced locally rather than derived from the central nervous system [26].

In melanocytes, CRH can trigger a succession of signaling cascades similar to the algorithm in the HPA axis: CRH and CRH-R1 interact together to increase the levels of cAMP and promote the release of POMC [24,27]. Then melanocytes utilize enzymatic machinery including 7B2 protein and proprotein convertase 1 and proprotein convertase 2 to process POMC into α-MSH and β-endorphin and ACTH; the latter in turn increases the synthesis of cortisol and corticosterone [21].

Remarkably, the neuroendocrine activities of melanocytes can be regulated by various internal factors, including cytokines, and growth factors, as well as external factors, including ultraviolet radiation (UVR), chemical and physical stimulus, which can all modulate hormone secretion and modify phenotypic activities [9,25,28,29]. It has been demonstrated that UV-B, an epidermal-specific stressor, can stimulate the CRH promoter and induce CRH production through protein kinase. The interaction of CRH with the CRH receptor 1 subsequently activates the POMC expression, followed by converting to ACTH, α-MSH, and β-endorphin [25]. UV-B exposure can increase intraepidermal β-endorphin production, which may explain, in part, why sun exposure and sunbeds can result in sun-seeking addictive behavior [25,30,31]. Melanocytes counteract environmental stressors following the same algorithm as the central stress response. Hence, it is proposed that this functional homolog of the HPA axis plays an indispensable role in the cutaneous sophisticated stress response network [27,29]. This similar activation pattern in response to stress also puts forward the hypothesis regarding the evolution of the stress system, which states that biological stress-coping systems may have originated from the integument [5,28]. Furthermore, it has been proposed that these cutaneous hormones not only play a crucial role in regulating local homeostasis but might also regulate global homeostasis by transmitting signals through blood vessels or nerves [28,32].

It is worth mentioning that melanocytic lineage cells, including nevus cells and melanoma cells, all express CRH mRNA and peptides, and their expression is enhanced during tumor progression [33,34,35]. Vitiligo is a cutaneous autoimmune disease of acquired pigmentary disorder, additionally, there are lower gene expressions of the melanocortin receptors (including MC1R and MC4R) and POMC in vitiliginous lesion skin compared to the nonlesional skin due to the loss of functional melanocytes [36]. Remarkably, though, an up-regulation of the melanocortin receptors in the unaffected skin of vitiligo patients was found compared with skin from healthy subjects, which could be implicated to be a compensatory response to increase the lesion pigmentation [36]. The melanocortin system in the skin, a part of the skin’s HPA axis, has been shown to act as a coordinator and executor of local stress responses by regulating the skin’s inflammatory and pigmentation networks. Therefore, changes in the melanocortin system in the vitiligo lesions could be related to the pathogenesis of vitiligo. Moreover, psychological stress is known to activate the HPA axis and aggravate vitiligo. Given that melanocytes play dual roles as prominent targets and sources of the peripheral HPA axis, a study assessed the possible relationship between CRH and CRHR-1 with psychological stress and found that the expressions of CRH and CRHR-1 in vitiliginous skin were positively correlated with psychological stress, which was assessed by a questionnaire [37]. Accordingly, this finding implies that psychological stress might have a direct effect on the local HPA axis, particularly CRH and related peptides, which may play a role in the occurrence and exacerbation of pressure-related vitiligo disorders [37]. Additionally, it has been observed that hydrogen peroxide (H_2_O_2_) mediated oxidation of the hormones of epidermal ACTH, α-MSH and β-endorphins in vitiligo, leading to the loss of their functions in promoting melanocyte pigmentation in vitiligo [38]. Thus, the redox balance of POMC-associated peptides might correlate with the onset of vitiligo. Consequently, further research should be conducted on the potential role of the neuroendocrine activity of melanocytes and the mechanisms by which they are regulated in physiological and pathological processes, which will contribute to the treatment of pigment disorders such as vitiligo.

### 2.2. Hypothalamic-Pituitary-Thyroid Axis (HPT) Homolog

In humans, thyroid gland disorders lead to several alterations in skin architecture and homeostasis [39]. The skin of hypothyroid individuals is often pale. In contrast, patients affected by hyperthyroidism present with diffuse skin pigmentation [40,41]. Indeed, human epidermal melanocytes (HEMs) have been reported to express several molecular elements of the hypothalamic-pituitary-thyroid axis (HPT), including TSH, TRH, and their corresponding receptors [9,42,43]. Moreover, the expression of epidermal TSH is upregulated by TRH and repressed by thyroid hormones, just like the central HPT axis is organized hierarchically. These TRH and TSH receptors detected in melanocytes have been demonstrated to be biologically active, which may offer a possible explanation for the phenotypic changes of melanocytes following TRH or TSH treatment [39,41,42,43]. In addition, the genes coding for sodium iodide symporter (NIS) and thyroglobulin (Tg) were expressed in epidermal melanocytes and deiodinases 2 and 3, which are responsible for converting T4 into T3 [42].

Vitiligo is frequently accompanied by thyroid disorders, and 34% of patients have positive thyroid antibodies [44]. The autoimmune hypothesis is the most widely accepted and holds that serum thyroid antibodies such as thyroglobulin antibody (TgAb) and thyroid peroxidase antibody (TPOAb) separately bind to thyroglobulin (Tg) and thyroid peroxidase (TPO) expressed in melanocytes, resulting in their activation, which may partially account for the destruction of the melanocytes in vitiligo [45]. Another fascinating hypothesis is that autoantibodies such as TgAb and TPOAb can induce sustained oxidative stress, which in turn leads to apoptosis and enhanced senescence of melanocytes [46]. Given the clinical correlation between these two diseases, vitiligo patients are suggested to routinely test thyroid antibodies [39,41,44].

In addition, TRH has been identified in melanoma cell lines and dysplastic nevi, and the expression of TRH is higher in dysplastic nevi and melanoma compared with benign nevi, thus implicating that TRH might be involved in the malignant conversion of melanocytes into melanoma cells in a paracrine or autocrine manner [47]. Moreover, it is important to emphasize that low-concentration TRH treatment leads to the proliferation of melanoma cells, an effect that could not be observed in cultured normal melanocytes [47]. Shortly thereafter, the same group found that melanocyte-originated lesions ranging from a benign nevus, dysplastic nevi, and melanoma all express functional TSHR, with an upregulated expression in premalignant and malignant lesions, implying a higher sensitivity to TSH [48]. Combining this with the study that found TSH can promote the growth of melanoma by triggering the formation of cAMP and activating the mitogen-activated protein kinase (MAPK) signaling pathway, may in part account for the high prevalence of hypothyroidism (elevated TSH lever) in the melanoma population [48,49]. Intriguingly, another study has reported that suppression of MAP kinase and PI3KAkt pathways exhibits their anti-melanoma effects, including suppression of cell proliferation, transformation, and invasion, and these effects are coupled with inducing the expression of thyroid genes, such as TSH-R and NIS, and consequently increasing radioiodide uptake by melanoma cells, which may prove to be a novel approach to treating melanoma [50,51].

### 2.3. Serotoninergic/Melatoninergic System

Melatonin (MT), an evolutionary ancient hormone, has been found in almost all organisms, ranging from animals, plants, and microorganisms [52,53]. Increasing studies have revealed that melanocytes can endogenously produce melatonin and its precursor serotonin (5 hydroxytryptamines: 5-HT) [54,55,56]. Key biosynthetic enzymes, including tryptophan hydroxylase (TPH), arylalkylamine N-acetyltransferase (AANAT), and hydroxy indole-O-methyltransferase (HIOMT), are responsible for the sequential metabolic conversion into 5-HT and melatonin [56]. These enzymes have been proved to be expressed and be enzymatically active in melanocytes [55,57].

Well established as the guardian of the genome and cellular and tissue in humans, melatonin exerts pleiotropic functions via melatonin receptor-dependent or non-receptor-dependent pathways such as potent antioxidative, free radical scavenging, anti-inflammatory, and mitochondrial protective activities [52,53]. It has been reported that melatonin and its metabolites, such as 6-hydroxymelatonin (6(OH)M), N1-acetyl-N2-formyl-5-methoxykynuramine (AFMK), can inhibit proliferation, pigmentation, and DNA synthesis in a dose-dependent manner in melanocytes [58]. Moreover, human melanoma cells have been experimentally validated to synthesize and metabolize serotonin and melatonin [54,55,59]. Furthermore, melatonin and its metabolites can modulate melanogenesis, ameliorate UVR-induced mitochondrial oxidative stress, and inhibit proliferation in melanoma cells [60,61,62]. In addition, accumulating evidence suggests that they can also mediate phenotypic actions, including regulating keratinocyte proliferation and differentiation, enhancing epidermal barrier formation, regulating hair growth cycling, and counteracting UVB-induced skin damage [60,63,64,65]. Hence, the topical application of these multifunctional molecules is promising and attractive in the treatment of pigmentary disorders and other skin diseases, such as atopic dermatitis [52,53,61].

### 2.4. Other Neuroendocrine Activities

Other examples of neuroendocrine activities that exist in melanocytes are sequentially synthesizing the catecholamines, which begins with L-tyrosine, followed by generating L-DOPA, dopamine, norepinephrine, and epinephrine through classic enzymes (e.g., phenylalanine hydroxylase (PH), tyrosine hydroxylase (TH), etc.) [66,67,68,69]. The activity of PH and TH, the rate-limiting step of catecholamine biosynthesis, relies on their functional cofactor, 6R-L-erythro-5,6,7,8-tetrahydrobiopterin (6BH4) [6,9]. Importantly, melanocytes are capable of de novo synthesis and circulating regulation of 6BH4 [66]. In particular, an important yet often overlooked neuroendocrine activity could be L-DOPA or its derivatives, which are intermediates produced in melanogenesis. It has been suggested that they can act as hormone- and neurotransmitter-like roles that may even enter into the circulation and subsequently coordinate global homeostasis [6,10,70].

In summary, these findings signal a new perspective on the pivotal role of melanocytes as neuroendocrine cells that receive, integrate, and transform environmental information into local and systemic effects. The latter may include neural or humoral responses to precisely maintain the local or even the whole body’s dynamic balance.

## 3. Melanocytes and Skin Immunity

The skin harbors a complex, coordinated system composed of various immune cells and epithelial tissue cells that defend against tissue invasion by pathogens, toxins, or physical injury. Moreover, emerging evidence has recently revealed that melanocytes also play an active role in the skin’s innate immune defense and adaptive immune response and have immunomodulatory properties [5,71,72,73].

### 3.1. Melanocytes Are Functional Immune Sentinel Cells in the Skin

Located strategically in the epidermis, melanocytes provide external barriers along with keratinocytes and Langerhans cells to eradicate pathogenic threats and maintain health [5,72]. Melanocytes are constantly challenged by potentially harmful pathogenic microbe stimuli for their unique position [74]. It has been proposed that HEMs can be infected by viral pathogens (e.g., *varicella-zoster virus* and *parvovirus*) and bacteria (e.g., *Leptospira* and *Mycobacterium leprae*) [72]. Therefore, it has been proposed that the onset of vitiligo is related to viral infection of melanocytes, and melanocytes are directly killed by viral infections (viral pathology) or are destroyed by immune-mediated autologous melanocyte damage (immunopathology) to limit virus expansion [75,76,77].

From another perspective, melanocytes should not be just seen as passive targets for pathogens, but instead immunocompetent cells, as their contribution to immunity defense is increasingly recognized [73,78]. Biologically, melanocytes express various immunologically active proteins and functional receptors and perceive the change in the barrier integrity, microbial invasion, and stress [5,72]. Thus, they act as sentinels that constantly deliver information to the cutaneous immune system [72]. Moreover, the dendritic structure of melanocytes and their wide distribution in the skin, combined with their strategic distribution in the epidermis, suggest that they perform essential immune functions in the skin’s immune defense system [5,72].

### 3.2. Innate Immune Responses

The essence of the immune response is to quickly limit the infection of the pathogen at the main invasion portal (such as skin) before its widespread transmission [79]. The first step to initiating an effective antimicrobial innate immune defense is to recognize potential pathogens and then respond appropriately to their intrusions while avoiding damage to surrounding cells [80]. To combat the invasion of pathogens, cells in the skin, including melanocytes, express many different pattern recognition receptors (PRRs), germline-encoded sensors best characterized for their vital role in host defense, which contain Toll-like receptors (TLRs), nucleotide-binding oligomeric domain-like receptors (NLRs), C-type lectin receptors (CLRs), and retinoic acid-induced gene 1 (RIG-I)-like helicase receptors (RLRs) [72,78]. These receptors have evolved to detect components, including pathogen-associated molecular patterns (PAMPs) present on foreign pathogens and damage-related molecular patterns (DAMPs) on host cells that do not appear under normal circumstances [72,78].

When invaded by extracellular bacteria (such as *spirochetes*) and intracellular viral pathogens (such as *alphavirus*), the skin melanocytes mobilize their innate immune function and initiate the inflammatory cascade [72,81]. Subsequently, melanocytes trigger signal pathways by transcription factors, including interferon regulatory factor 3 (IRF3), IRF7, nuclear factor-κB (NF-κB), and activator protein-1 (AP-1), and then promote the expression of type-I interferons (such as IFN-α and IFN-β), chemokines (such as CXCL-8, CCL-2) and various pro-inflammatory cytokines [80,82]. Furthermore, the IFNs can amplify the reaction and mobilize adjacent uninfected cells, which will further stimulate the expression of numerous IFN-stimulating genes (ISGs), pro-inflammatory cytokines, and chemokines, leading to a robust antimicrobial effect [72]. In addition, melanocytes have been proven to represent the first line of innate immunity to resist fungal pathogens such as Candida albicans [83].

Several experiments have found that stimulation of normal human melanocytes with lipopolysaccharide (LPS) significantly enhanced the expression of TLR4 and secretion of numerous inflammatory cytokines and chemokines, such as interleukin (IL)-1β, IL-6, IL-10, IL16, TNF-α, CCL2, CCL20, CXCL8, and CXCL12 [84,85]. Intriguingly, noticeable differences in the expression of inflammatory mediators were found between melanocytes derived from dark and light skin after stimulation with LPS, indicating a relationship between the immune properties and the pigmentation phenotype of normal melanocytes in vitro [84]. It was also found that repeated UVR induces the expression of TLR4 and increases the secretion of IL-6 and IL-10 in neonatal human melanocytes, suggesting that melanocytes may have a role in UV-induced immunity modulation [86].

There is a close link between melanin synthesis and immunity [87]. Melanization acts as a protective barrier to inhibit the growth of pathogenic microorganisms, as many toxic quinone intermediates formed in the process of melanin biosynthesis are shown to possess potent antibacterial and antifungal properties [71,88,89]. It has been demonstrated that melanin, the final product of melanization, has a strong binding property to physically capture invading microorganisms and then inhibit proliferation and can neutralize bacteria-derived toxins [71,90]. Therefore, Montefiori et al. proposed the possibility of treatment for acquired immune deficiency syndrome (AIDS) by using soluble melanin to inhibit HIV replication [91]. Moreover, melanosomes, melanin-producing organelles, contain numerous lysosomal enzymes which can degrade bacteria and other tissues [89]. In addition, the melanosomes transferring from melanocytes to keratinocytes may acidify the stratum corneum, while this acidic pH of the stratum corneum, called “acid mantle,” can provide an unfavorable condition for certain microorganisms, which is vital for skin barrier function and microbial defense [92,93]. There is a hypothesis that barrier requirements are the evolutionary “driving force” of human ancestor epidermal pigmentation, which supports skin structure and function [94,95]. Additionally, from an evolutionary perspective, skin pigmentation is a product of natural selection to resist varying degrees of UVR [96]. Although skin pigmentation has additional benefits, such as energy transfer, radical scavenging, and camouflage, it is essential to produce an effective permeability barrier [97]. It should be emphasized that melanization has long been considered to be a canonical and major component of the invertebrate defense system [3,71,89]. It is also worth noting that people with fair skin tend to be more susceptible to skin infections than darker-skinned individuals clinically [71,89]. The skin of the stable and noninflamed vitiligo lesion sites showed an inferior barrier function than the adjacent normal skin sites of the same individual [98]. Finally, it is also worth mentioning that TLRs play essential roles in regulating melanogenesis in microbial- or inflammation-related processes. It was demonstrated that the activation of TLR2, TLR3, TLR4, and TLR9 increased melanogenesis in melanocytes, while stimulation of TLR5 and TLR7 inhibited melanogenesis [87,99,100].

### 3.3. Adaptive Immunity

Melanocytes are capable of phagocytosis, antigen processing, and presentation in which the melanosomes contain sufficient enzymes needed for processing the antigen [101]. Meanwhile, melanocytes can express various immunological marks, including major histocompatibility complex class II molecules (MHC-II) [102]. Combined with their dendritic nature combined with their strategic location and phagocytosis activities [102], human melanocytes have been demonstrated to process and present antigenic peptide fragments and intact protein preparations to CD4+ cytotoxic proliferative T-cell clones in an MHC class II-restricted and antigen-specific manner, suggesting that melanocytes serve as non-professional antigen-presenting cells [103].

Melanocytes can also express several important marker proteins, including intercellular adhesion molecules 1 (ICAM-1), CD40, VCAM1 and HLA classes I and II, which are essential for the co-stimulation of T cells [104]. ICAM-1 mediates non-antigenic cell contact, which plays a key role in lymphocyte migration, activation, adhesion, cell and antibody-dependent cytotoxicity, while CD40 is essential in T-cell-dependent B cell activation, proliferation, and differentiation [72]. After CD40 binds to its ligands, melanocytes are able to upregulate their co-stimulating and adhesion molecules on their surfaces, suggesting that melanocytes have immune function [72].

**Figure 1:**
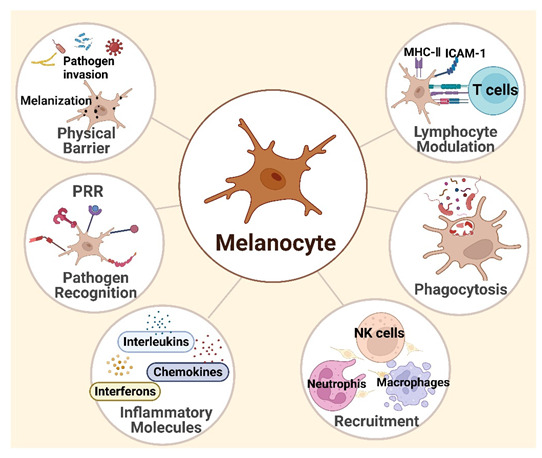
Melanocytes in skin immunity. Melanocytes located strategically in the epidermis act as an external barrier to pathogenic threats. They express many different pattern recognition receptors (PRRs) and mobilize their innate immune function and initiate the inflammatory cascade after activating PRRs. Subsequently, melanocytes trigger signal pathways and secrete interferons (such as IFN-α and IFN-β), inflammatory cytokines, and chemokines, such as interleukin (IL)-1β, IL-6, IL-10, IL16, TNF-α, CCL2, CCL20, CXCL8, and CXCL12. On the other hand, melanocytes are capable of phagocytosis of pathogens, then processing and presenting antigen to CD4+ T-cell clones in an antigen-specific and MHC class II-restricted manner, indicating that melanocytes function as nonprofessional antigen-presenting cells. The figure was created using BioRender.

In summary, a large body of evidence supports HEMs as not merely professional melanin-producing cells but also significant contributors to the skin’s immune defense as immunocompetent cells, as shown in Figure 1.

## 4. Melanocytes and the Sensory System

The capability to sense and respond to an ever-changing environment is a prerequisite for the survival of organisms [105]. Classical studies have mapped out our understanding that sensory receptors are confined to be present in the initially identified sensory cells: opsin proteins are found in retinal sensitive cells and are activated by light at specific wavelengths, serving as a common molecular basis in animal light reception [105,106,107]. Olfactory receptors (ORs) are primarily expressed in the cilia of the olfactory epithelium in the nasal cavity, responsible for perceiving chemosensory signals and resulting in olfactory input [108].

In addition to exerting classical functions in the nose and eyes, growing evidence has suggested that opsins and olfactory receptors are also expressed in numerous types of skin cells, including melanocytes, involved in regulating diverse biological processes such as hair growth [109,110], wound healing [111,112], photoaging [113,114], and melanin synthesis [115]. Consequently, the skin has been suggested to have evolved its sophisticated signaling transduction system and act as a window to perceive and respond to various environmental cues [106,116].

Here, we highlight the current perceptions about the sensory receptors found in skin melanocytes, focusing on recent breakthroughs and their local and systemic effects.

### 4.1. The Melanocyte Photosensory System

Light is an important environmental signal for organisms from all kingdoms of life. The skin and the eye are both constantly exposed to light stimuli [106]. Recently, numerous studies have shown that skin also possesses a wide variety of opsins that sense light to regulate skin physiological processes accordingly and participate in circadian entrainment in peripheral tissue, greatly expanding our knowledge of opsins beyond their recognized role in the eyes [106,117].

Opsins can be primarily divided into two large groups: (i) classical visual opsins, including cone opsins (opsin1; OPN1) and rhodopsin (opsin 2; OPN2); and (ii) non-visual opsins, including panopsin (opsin 3; OPN3), melanoprotein (opsin 4; OPN4), and neuropsin (opsin 5; OPN5). Cone opsins are well-known photoreceptors found in the cone cells of the retina and detect light⁄dark contrasts. Rhodopsin is also a photosensitive protein present in the rod cells of the retina, which underlies color vision [106,117,118]. Besides the classical visual opsins, it has been considered that several other opsins are also important for non-image-forming visual processes [106,118]. Likewise, present in the retina, melanopsin (OPN4) is considered to mediate circadian photoentrainment, pupillary light reflex, and melatonin suppression [107]. Neuropsin (OPN5), expressed in several tissues including the brain, is involved in regulating seasonal reproductive behavior in birds and rest-activity cycles in mice [118]. In addition, OPN5 is thought to contribute to the local circadian oscillators’ photoentrainment of the retina and cornea in mammals [119]. The remaining opsin, OPN3, was properly named encephalopsin for its initial identification in the brain. Later, it was discovered in other tissues and aptly renamed panopsin [120]. All these photoreceptors respond to specific wavelengths of light through protein-coupled receptors (GPCRs) to trigger signaling transduction cascades [105,118].

#### 4.1.1. Phototransduction

The earliest evidence of opsin in the skin was reported in the dermal melanophores of *Xenopus laevis* where OPN4 mediates light-induced chromophore dispersion, hence adjusting the skin color shade [121,122]. *Xenopus laevis* has been widely used as a model organism for cellular and developmental biological research, and it was also later shown that the melanophores of amphibians are homologous to the melanocytes of mammals [123,124]. From then on, the concept of skin photoreception has been proposed, and increasing studies have demonstrated that opsins, key light-detecting proteins in the retina, are identified as photosensors in the skin across many nonhuman animals and humans [117,118,125,126,127]. In nonhuman animals, opsin or opsin-like proteins have been found in the skin of fruit flies, zebrafish, frogs, octopuses, and mice, and they are involved in behaviors that are critical for survival, such as regulating skin color, light avoidance, shadow reflexes, and circadian photoentrainment [107,117,118]. Opsins are expressed in various human skin cell types, including melanocytes, and are involved in various physiological processes [117,118].

In 2011, Wicks et al. first found that UVA evokes retina-dependent calcium fluxes in HEMs, suggesting this effect might be mediated by an opsin-like photopigment. Subsequently, rhodopsin was detected in melanocytes and was further proved to contribute to UVR-induced Ca^2+^ signaling. To test whether rhodopsin-mediated light transduction regulates melanin production, this team compared melanin production in rhodopsin-targeted expressing or control miRNA groups, although no significant variation was found, likely due to the sustained Ca^2+^ responses caused by residual rhodopsin expression. Conversely, when mimicking knockdown of opsin receptors by excluding retinal, the researchers found HEMs treated with 9-cis retinal exhibited a significant UVA-induced melanin increase compared with HEMs without retinal, identifying a new UVA-sensitive signaling pathway in melanocytes that stimulates Ca^2+^ mobilization and drives immediate pigment darkening (IPD), resembling the well-characterized phototransduction cascades in the eye [128]. Later, other researchers observed a multitude of opsin receptors expressed in HEMs, including OPN1-SW, OPN2, OPN3, and OPN5; among them, OPN2 and OPN3 were significantly more abundant [129]. The expression of opsins in the skin suggests that these proteins may exert their functions alone or synergistically in mediating phototransduction pathways in human skin, enthusing the enhanced curiosity of researchers about the nature of this exciting photo-sensory system.

A further study proved that OPN3 is the key sensor responsible for visible-light-induced pigmentation and demonstrated that melanogenesis induced by OPN3 is calcium-dependent, further activating Ca^2+^/CaMKII/CREB signaling pathways, leading to the enhanced phosphorylation of MITF and ultimately upregulating melanogenesis-associated enzymes: tyrosinase and dopachrome tautomerase (DCT), whereas such an effect is abrogated by silencing OPN3 [130,131]. Literally, HEMs can see the light through the OPN3 sensor and respond by inducing a potent and long-lasting pigmentation, suggesting OPN3 could be an emerging target candidate for pigmentary disorders. Since then, other studies have also shown that OPN3 may be crucial in the regulation of melanin production but have proposed a novel insight into the mechanism of action of OPN3 [132]. They demonstrated that OPN3 negatively regulates melanogenesis via the Gαi pathway, inhibiting melanocortin 1 receptor (MC1R) and thus modulating melanin levels in human melanocytes [132]. In addition, another study indicated that downregulating OPN3 significantly decreases the intracellular calcium content in melanocytes and leads to conventional apoptosis via a mitochondria-mediated pathway [133]. Furthermore, it has also been reported that melanopsin and rhodopsin are sensors of UVA radiation in normal and malignant murine melanocytes, mediating immediate pigment darkening through calcium and cGMP pathways [134]. Interestingly, UVA-driven IPD is fully and completely abrogated upon knocking down OPN2 or OPN4 by siRNA or pharmacological blockade by AA9253 [134]. Although several previous reports have suggested that human skin cells do not express OPN4 [128,129], a recent study challenged this perspective by showing that OPN4 is expressed in human skin tissues and isolated melanocytes [129]. Furthermore, it has been shown that OPN4 is involved in mediating calcium influx and downstream ERK activation following blue light irradiation similar to the retina and is suggested to belong to the skin photosensitive system [129]. There is also evidence of OPN5 involvement in sensing and phototransduction in HEMs, with it being further proved that OPN5 is the important sensor relating to UVR-induced pigmentation through triggering the calcium-protein kinase C (PKC) signaling cascade and activating MITF and thus contributing to upregulating melanin production [115].

Therefore, light signals are converted into intracellular signals through OPN in human skin photoreception, and this new phototransduction mechanism is of vital importance for understanding photobiomodulation (PBM) therapy, a form of local light therapy based on non-ionizing light sources. Clinically, PBM therapy is becoming a promising treatment for a variety of skin conditions, such as hair regeneration, wound healing, psoriasis, eczema, and atopic dermatitis [135,136,137,138,139]. Future studies focusing on elucidating the underlying mechanisms of different opsins to regulate skin physiological processes, and defining the corresponding light parameters to optimize light therapy will expand clinical indications for PBM and change the treatment landscape for more skin diseases.

#### 4.1.2. Circadian Photoentrainment

Life on earth evolved in an environment of a predictably changing cycle of light and darkness. Organisms keep track of daily and seasonal environmental changes by perceiving ambient light cues and have been equipped with an endogenous timekeeping system, the circadian rhythm, to predict and accommodate these recurring environmental changes [140]. Virtually almost all mammalian cells and tissues possess a biological clock with a free-running period that must remain synchronized (entrained) to the daily environmental cycle of 24 h.

In mammals, light and dark (LD) cycles are vital environmental timing cues, the first Zeitgeber [140,141]. The synchronization of an organism’s endogenous rhythms to an exogenous LD cycle is termed photoentrainment. External light stimulus is decoded by specialized OPN4-expressing cells, the intrinsically photosensitive retinal ganglion cells (ipRGCs), which convert the light information into an electrical signal and transmit it to the central clock localized in the suprachiasmatic nucleus (SCN), which then transmits neural or hormonal messages to synchronize the peripheral organs [107,140].

Previous studies have provided strong support for the presence of a peripheral circadian oscillator clock in the mouse skin [141]. Subsequently, the existence of clock genes and functional oscillators was reported in human skin, including primary human melanocytes as well as keratinocytes and dermal fibroblasts [142]. Additionally, it has been suggested that skin, an operationally easy tissue, is an ideal sample source to determine the body-clock time [143].

Numerous studies in the past two decades have strongly suggested that opsins in the retina play a special role in the photoentrainment of mammalian circadian rhythms [106,107,119]. In particular, the opsin family evolved prior to the evolution of image-forming visual systems, suggesting that the original role of opsins was nonvisual, including checking time-of-day information based on photoperiod, intensity, and wavelength of light [144].

Recent research has demonstrated that neuropsin (OPN5), previously known to mediate photoentrainment of the circadian oscillator in retinal neurons [119], is also expressed in murine skin melanocytes and exerts its function locally in a light-sensitive mechanism to photoentrain circadian rhythms and then stimulate the expression of clock genes [145]. Using the *Opn4^−/−^;Pde6b^rd1/rd1^* blind mice model, researchers further reported that peripheral opsin expressed in murine melanocytes is capable of substituting for the SCN in providing local photoentrainment signals in vivo, which allows the dermal circadian oscillator to synchronize to the day-night cycle even when the central clock is free-running [145].

Moreover, numerous studies have revealed that melanocytes have a photosensitive system to decode and respond to light information and play a unique role in the photoentrainment of circadian rhythms. De Assis et al. explored the influence of white light pulse (WLP) on normal and malignant murine melanocytes and found that WLP does activate the clock gene machinery in malignant melanocytes with marked upregulation of core clock genes Per1, Per2, and Bmal1 [146]. Additionally, investigators found that malignant melanocytes display increased sensitivity and elevated responses to WLP compared with normal melanocytes, with a lower expression of Opn4 [146]. Hence, the researchers suggested the possibility that the malignant transformation of melanocytes might modify the intrinsic photosensitive system [146]. Subsequently, the same team further explored the unique function of Opn4 on responses to UVA radiation, including regulation of proliferation, apoptosis, pigmentation, and molecular clock, and found that UVA-induced effects are abrogated in murine normal melanocytes and Opn4 absence melanoma cells [147]. Additionally, it has also been found that the expression of OPN4 decreases in human melanoma, and its downregulation is significantly associated with tumor metastasis and poor prognosis [147]. Besides the classical light receptor function, melanopsin has also been demonstrated to mediate the heat activation of murine clock genes (Per1 and Bmal1) and acts as a thermoreceptor that ultimately regulates the local circadian clock [148]. Here, it is worth mentioning that the temperature is also a circadian clock timer [140,148].

Reptiles form an apical eye on the top of their heads that senses light–darkness [149]. Several photoreceptors are found in the pineal glands of chickens and mice [150,151]. In humans, however, the pineal gland is located in deeper regions of the brain. On the other hand, it has been shown that exposure to bright light can affect the circadian rhythm of blind patients [152]. Likewise, light pulses exposed to the popliteal region (behind the knee) were shown to affect the circadian clock of healthy individuals [153]. These findings challenge the traditional cognition that mammals are incapable of extraocular circadian phototransduction and have implied the presence of a light-sensitive system on the body’s surface which generates time-related signals in mammals [153]. The central circadian pacemaker within the mammalian brain is commonly considered to dictate the rhythms of peripheral organs; however, various studies have implied the presence of a direct light-response mechanism and a local photoentrainment pathway in mammals, similar to pathways in invertebrates and lower vertebrates [140]. As mentioned, reptiles utilize the parietal eye as a photoreceptor, which disappeared during evolution [128,141,150]. Consequently, it has been proposed that a photosensitive system may have evolved in mammal skin to compensate for parietal eye loss [127,144].

Taken together, melanocytes, as photosensitive cells in the skin, generate a photosensitive system by expressing several opsins responding to ambient light and play an important role in the regulation of peripheral circadian rhythm, as well summarized in Table 1. The functions of various opsins existing in the skin should be further investigated to gain insight into the evolution of the circadian rhythm of animals and understand the essence of the photo-sensory system and its implications for skin physiology.

### 4.2. The Melanocyte Olfactory System

Olfaction is the principal chemosensory system in many organisms and detects diverse smells [105,108]. Odor perception starts with odorant molecules binding to specific olfactory receptors (ORs), which are primarily expressed in olfactory sensory neurons, and then proper chemical signals are transmitted to the brain via electrical signals [105,108]. Numerous reports have described that ORs are also ectopically expressed in multiple extra-nasal tissues. These ORs have generally not been thought to have any significant impact on the pathology of any common disease and have limited potential as therapeutic strategies. Nevertheless, this viewpoint is drastically and rapidly changing as increasing ectopic ORs, responding to various ligands, have been reported to be involved in distinct biological processes including sperm chemotaxis, wound healing, hair growth, muscle regeneration, tumor suppression, and energy metabolism [109,154,155,156,157,158].

The skin is the outermost barrier of the human body and is regularly exposed to multiple environmental chemical stimuli. Several ORs have been identified in diverse cell types housed in the skin, including melanocytes and keratinocytes [154,155,159,160,161]. The expression of diverse cutaneous chemical-sensitive receptors contributes to decoding environmental odor information [116,159]. The developing olfactory system originates from both the embryonic ectodermal placode and the neural crest [162]. Intriguingly, ORs have also been found in keratinocytes and melanocytes that precisely originate from surface ectodermal cells and neural crest cells during the embryonic period, respectively [163].

In the 1980s, the first evidence of the effects of odor on pigment cells was presented in frog melanophores dispersing their melanosomes in response to the same chemical class and concentrations of odorants used in olfactory cilia studies, and this process was correlated to increasing intracellular cAMP [164]. At that time, the superfamily of odor receptors was still unknown. Corresponding studies found melanosome dispersion in fish melanophores responding to the stimulus of cinnamaldehyde and β-ionone [165].

Later, the expression of OR51E2, an olfactory receptor which is located in the olfactory epithelium, was also detected at the transcript or protein levels [161]. It has been further demonstrated that activation of OR51E2 by a specific agonist, β-ionone, ultimately inhibits melanocyte proliferation and stimulates melanogenesis [161]. It is possible that β-ionone, commonly found in beauty care products, may contribute to cosmetic-induced hyperpigmentation [161]. Soon afterward, OR2A4/7 was verified to be expressed and play an active role in primary human melanocytes. The odorant cyclohexyl salicylate, a specific ligand of OR2A4/7, has been shown to inhibit proliferation and promote cell differentiation in conjunction with enhanced pigmentation in melanocytes by elevating intracellular cAMP and Ca^2+^ levels [162]. Together, these findings imply that several ORs may be potential targets for pigmentation disorder treatment [161,162].

In addition, as OR expression is upregulated in several cancer tissues, the promising application of ORs in cancer diagnosis and therapeutics has also emerged [108]. In particular, OR51E2 serves as a potential biomarker for melanoma [108]. The previous study identified that human melanoma expresses the olfactory receptor 51E2 and further showed that activating endogenous OR51E2 in cultured cells isolated from metastatic and vertical growth phases can suppress the growth of melanoma via inducing apoptosis [166]. In addition, research evaluating the expression profile of olfactory receptors in a multitude of cancer cell lines has shown that OR2C3 is exclusively expressed in melanoma lines [167]. Remarkably, the expression of OR2C3 was confirmed in human melanoma cells but in not normal melanocytes [167]. Therefore, the pattern of OR2C3 expression implies a potential role in the initiation and/or development of melanoma [166,167].

Thus, human melanocytes can “smell” in the sense that they recruit the evolutionarily ancient and largest of receptor families to regulate skin pigmentation, cell proliferation, apoptosis, and tumorigenesis. Although informative, most of the insights into ORs’ role come from studies performed in vitro cell-based models, leaving several biologic questions unanswered. The expression levels of OR transcripts are low; therefore, further confirmation of the function of these ectopic ORs in vivo is urgently needed. Furthermore, it is critical to confirm the role of these receptors in humans. For verifying functions, especially in vitro, most experiments rely on ligand-induced activation of ORs, and few ligands have been identified. The molecular basis of the specificity and sensitivity of ORs is poorly understood. Therefore, the lack of knowledge of the ligands of ORs is an obstacle that needs to be overcome for future research. Furthermore, the identification of endogenous ligands is challenging, and humans may not always share the same ligands with mouse receptors. Moreover, odorant levels sufficient to sustain chronic excitation of the receptor are also worthy of consideration for future clinical applications. In addition, are melanocytes able to transmit signals to the brain like olfactory epithelial cells after sensing an odor? Although the current understanding of the biological function, signaling cascades, and pharmacology of ectopic ORs remains insufficient and problematic and may represent merely the tip of the iceberg, future in-depth exploration and recognition of the potential roles of ORs may lead to the use of potential odorants as a promising strategy for the treatment of various diseases based on the theory of ectopically expressed ORs.

## 5. Melanocytes Outside the Skin

Melanocytes predominantly inhabit the skin epidermis, hair follicles, and the iris of the eye, where they have various functions including producing melanin for protection from ultra-violet radiation and are responsible for the coloration of the skin, hair, and eyes in animals, which is of crucial importance in various aspects of the animal’s life, including communication and visual camouflage [1]. Meanwhile, melanocytes play vital roles in embryonic development and organ functions, as can be seen in patients with oculocutaneous albinism type 1 (OCA1), which is caused by mutations of the TYR and leads to hypopigmentation in the skin, hair, and eyes, consequently bringing actinic damage and a higher risk of skin cancer [168]. Most melanocytes originate in neural crests, follow diverse and interesting developmental pathways, and finally migrate to specific areas of the developing embryo. Interestingly, melanocytes are also located in deeper parts of the body that are not directly exposed to UVR, including in the striatum vessels of the inner ear, in the substantia nigra, leptomeninges, and locus coeruleus of the brain [163]. In addition, these extracutaneous melanocytes are present in other lesser-known regions, including cardiac valves, the septum, and major arteries and veins in the heart. Therefore, this begs the question of what key roles do these melanocytes in such a sun-protected habitat play in still producing melanin? Here, we briefly summarize the function of the extracutaneous melanocytes, primarily focusing on the heart and ears to observe the structural significance they contribute.

### 5.1. Heart

The presence of pigment cells in the heart was first mentioned in a PET⁄MCV mouse strain, although it could not clearly indicate their origin or whether they were melanocytes [169]. More recently, Mjaatvedt et al. discovered a group of pigment cells located in the atrium, mitral valve, tricuspid valve, and ventricular septum in fetal and adult C57BL/6J mouse hearts [170]. These cells are positive for tyrosinase-related protein 1, a specific marker of melanocytes and their precursors [170]. Subsequent studies have further described cardiac pigment cells. These cells also express dopachrome tautomerase (Dct), which is a recognized marker for melanocyte stem cells and relies on the same signaling pathways involved in normal skin melanocyte development [171]. These facts indicate that pigmented cardiac cells are melanocytes or melanocyte-related [171]. In addition, the cardiac pigmentation levels may be relevant to coat color [172]. It has been repeatedly proven that melanocytes are located in the valves (mitral, tricuspid, and aortic) and septum (ventricular and atrium) [171,173,174]; however, a distinct perspective has recently been put forward [173], proving the existence of melanocytes in the pulmonary valve and suggesting that the incidence and degree of pigmentation in the heart did not differ statistically with the coat color of the animals.

Concentrating on the atrioventricular valve, Balani et al. found that the presence of melanocyte pigmentation may affect the stiffness of the tricuspid valve leaflet of mice [174]. The viscoelastic property of atrioventricular valves is correlated with the degree of pigmentation. Furthermore, a novel finding supporting this view showed that the melanocytes of the atrioventricular valve mainly exist in the region that highly expresses versican B, a molecule responsible for the mechanical properties of murine atrioventricular valves [175]. Additionally, melanocytes may influence other extracellular matrix molecules to contribute to the changes in the valve microenvironment [175].

Intriguingly, cardiac melanocytes are considered to trigger atrial arrhythmias [176]. Both mouse and human cardiac melanocytes express the melanin synthase Dct, which is involved in buffering calcium and reactive oxygen species. Compared with wild-type mice, mice lacking functional Dct display increased susceptibility to atrial arrhythmias and are proven to produce frequent intracellular calcium oscillations. In contrast, mice lacking melanocytes in the heart fail to show atrial arrhythmia even if they lack DCT as well. Treating Dct-deficient mice with antioxidants can also significantly reduce arrhythmogenesis [176]. In addition, Hwang et al. showed cardiac melanocytes were located in the anatomical region, including pulmonary veins, atrioventricular valve annulus, left posterior atrium, and oval foramen, where atrial arrhythmia commonly occurs [177]. Then they confirmed that Dct expressed by cardiac melanocytes is involved with regulating whole atrial oxidative stress which leads to electrical and structural remodeling and triggers the occurrence of atrial arrhythmias in mice [178]. A further study uncovered the underlying mechanisms of the atrial arrhythmia hypothesis [179]. It is assumed that Dct−deficient mice may have upregulated small conductance calcium-activated potassium current and therefore may be more prone to initiating atrial arrhythmias [179].

### 5.2. Ear

Although generally localized in visible anatomical sites, melanocytes also exist as intermediate cells in the stria vascularis of the cochlea in mammals and are essential for proper cochlear development [180]. Melanocytes are also essential for normal auditory function by maintaining the endolymphatic potential at the scala media [180]. Additionally, numerous pericytes and perivascular macrophage-like melanocytes (PVM/Ms), as specialized melanocytes expressing several melanocyte markers, are known to play vital roles in stabilizing the intrastriatal fluid–blood barrier and sustaining the endocochlear potential, which is critical for normal hearing [181]. Moreover, these specialized melanocytes mediate disruption of the blood barrier responding to acoustic trauma, and finally result in the pathological deterioration of ear health after noise damage [182].

Hearing impairment, in some cases, is associated with genetic pigment disorders, such as Waardenburg syndrome type 2A (WS2A), a rare inherited disorder with autosomal dominant inheritance [183]. WS2A is caused by the mutation of microphthalmia-associated transcription factor (MITF), an essential regulator for melanocyte lineage development, and is characterized by varying degrees of pigmentation abnormalities and sensorineural hearing impairment [183]. There are other diseases exhibiting deafness or arrhythmias combined with hypopigmentation that further highlight the importance of the melanocytes, such as Alezzandrini syndrome, Vogt–Koyanagi–Harada disease, and Tietz syndrome [184,185,186]. Moreover, observations that bilateral cochlear dysfunction is common in both segmental and nonsegmental vitiligo individuals have illustrated the important role of melanocytes in cochlear function [187,188]. Additionally, it is suggested that vitiligo patients should take extra precautions to prevent acoustic trauma and avoid ototoxic drugs, especially during the period of disease activity, as their auditory system may be more vulnerable due to impaired melanocytes [187,188]. Cochlear dysfunction may increase with a longer duration of the disease; therefore, related audiometry tests should be performed for early detection of outer hair cells injury [189].

Interestingly, pigmentation is not essential for the hearing ability of the inner ear, as hearing ability is indeed largely unaffected in most albino mice and albino guinea pigs [180]. Nevertheless, it has been suggested that melanogenesis seems to exert protective effects in response to stressful conditions, including intense noise exposure and ototoxic injury [180]. The pigmented cochlea shows lower susceptibility to noise, which can be explained by the role of melanin as a scavenger of reactive oxygen species. In addition, the melanin of melanocytes in the inner ear is capable of incorporating drugs, such as aminoglycoside antibiotics and cisplatin, which also explains the potential toxicity to the auditory function of the cochlea [190,191,192,193].

### 5.3. Other Organs

In addition, other extracutaneous pigment cells, such as choroidal melanocytes, have been proven to contribute to visual function by supporting normal morphogenesis and maintaining functional vasculature structures and may not depend on their melanin-producing role [194]. There is evidence that the melanocytes in the brain might have neuroendocrine functions [195]. There is also evidence that the ectopic synthesis of melanin in human adipose tissue has been speculated as a compensatory mechanism for abating oxidative stress and responding to inflammation [196,197]. The functions of extracutaneous melanocytes located in various regions are summarized in Table 2.

Recent evidence has led to a renewed appreciation for the embryonic origin of cutaneous melanocytes in that these pigment cells not only originate from the migratory neural crest cells but also arise from the nerve-derived multipotent Schwann cell precursors (SCPs) [201,202]. Furthermore, this SCP-dependent origin of skin melanocytes has been conserved among fish, birds, and mammals during evolution [201,202,203,204]. Still, the origin of extracutaneous melanocytes located in the inner ear, brain meninges, heart, and other locations has been an “enigmatic” problem, fascinating scientists. Recently, this long-standing problem has been gradually addressed by a lineage-tracing strategy combined with 3D visualization, revealing that peripheral nerve-derived SCPs are an essential cellular origin of extracutaneous melanocytes [204,205]. The origin of such extracutaneous melanocytes can be associated with their localized specialization and their unconventional roles. But this complicates our understanding of what melanocytes really do [204]. Therefore, melanocytes outside the skin also exhibit fascinating functions other than melanin synthesis, and further exploration of the embryonic development of melanocytes will lead to a better understanding of the specific skin disorders and envisioning future prevention and treatment strategies [204].

## 6. Challenges and Future Perspectives

A critical re-examination of melanocyte biology provides new perspectives on the slowly emerging complexity of the physiological functions of melanocytes. Despite the great progress that has been made in the past few decades, there are still considerable thought-provoking problems and challenges yet to be definitively answered by future research.

### 6.1. Neuroendocrine Functions of Melanocytes in Health and Disease

The hormones regulating skin homeostasis have attracted increasing scientific and clinical attention. Though the effect of circulating hormones on skin has been well studied, it remains unclear in what manner and to what extent hormones locally synthesized by melanocytes exert effects in regulating other local and systemic physiologic activity. Moreover, the pathogenicity and clinical relevance remain to be completely elucidated. It is worth mentioning that an elegant study has directly proved the local synthesis of glucocorticoids in keratinocytes plays a broad role in the epithelial barrier by introducing an inducible knockout model with keratinocyte-specific deletion of Cyp11b1, which is the critical enzyme for de novo synthesized glucocorticoids [11] and has demonstrated that abrogation of local glucocorticoid synthesis activity can aggravate contact hypersensitivity, promote pruritus, and exacerbate psoriasiform inflammation [206]. In future research, therefore, conditional knockout of the Cre-Lox system under the melanocyte-specific promoter will be essential to study the important role of neuroendocrine activities of melanocytes in maintaining skin homeostasis in various physiological and pathological situations.

### 6.2. Multi-Directional Communication Network and Signaling

The “brain-skin axis” theory has been well described and contributes to explaining the underlying mechanisms of how psychosocial stress affects the homeostasis of healthy skin [207,208]. The skin and brain have close bidirectional anatomical and functional communication and share several identical hormones, neurotransmitters, and neuropeptides that mediate system stress responses. Anatomically located in the basal layer of the epidermis close to nerve endings and blood vessels, it has been proposed that melanocytes control the neuroendocrine activity of the epidermis and function as an amplifier of local sensation and transmission [79]. Therefore, it would be interesting and meaningful in future efforts to explore the interactions between melanocytes, the epidermis, dermis, and internal organs as well as brain and endocrine organs. Further research uncovering detailed mechanisms of how these local neuroendocrine signals transmit, amplify, and even integrate with other distal tissues will also add insight into the holistic management of stress-responsive cutaneous diseases including acne, psoriasis, alopecia, and vitiligo.

### 6.3. Neuroendocrine-Immune Interactions

Melanocytes are active participants in controlling the elimination of microbial infections and inflammation. However, our understanding of how these pathways subsequently lead to a strong immune response or even to a possible autoimmune response is still in its infancy. In addition, the cutaneous neuroendocrine system and the immune system are closely connected. As neuroendocrine-immune cells, melanocytes should be able to efficiently process and integrate the signaling exerted by the inflammatory and hormonal systems [209]. Exploring the orchestrated integration of the neuroendocrine system and the immune system in melanocytes will deepen our understanding of their function in sustaining homeostasis in a more comprehensive sense.

### 6.4. Circadian Rhythms and Neuroendocrine Pathways

Skin circadian clocks control various physiological processes, such as response to UV, wound repair, and immune response. Photoentrainment of the circadian clock is considered to be the basis for the stable regulation of neuroendocrine systems, leading to the secretion of hormones into the temporal ecological niche to better regulate physiological functions, including sleep, metabolism, and immune systems. Melatonin, ACTH, and cortisol, whose systemic fluctuations are controlled by the central pacemaker, are also locally produced in melanocytes. Thus, one future challenge is to investigate whether the local production of these hormones and their receptors is regulated by the peripheral circadian rhythms within the melanocytes and how these multiple cascades ultimately affect skin physiology.

### 6.5. The Intertwining of Light and Hormones

Besides the eye, the skin is the organ most widely exposed to sunlight as well as artificial light sources. Interestingly, the exposure of light to the skin has also been shown to not only exert local effects but also modulate cognition, memory, and emotion and produce different systemic effects, such as β-endorphins and urocanic acid production [210,211]. Current research suggests that OPN could be a candidate photo-receptor in the skin for mediating various light-dependent physiologies. Both skin and eyes are exposed to light [145]. Hence, the coming challenge lies in exploring whether the signals from skin and eyes reach the same coordinating central brain network and determining which neural pathways are activated and translated into neuroendocrine-immune effects. In this context, it is worth mentioning that the skin is considered to be a complex organ, with a peripheral neuroendocrine-immune structure that communicates with one another in a coordinated manner, and that this orchestrated algorithm of homeostatic regulation has been adopted and refined by the central neuroendocrine system in the course of evolution. Therefore, we have entered a new and exciting era of endocrinology research, perhaps best called “photoneurendocrinology”. Studies on neuroendocrine pathways activated by light exposure to the skin can open doors to unexpected and multifaceted biological correlations and uncover previously unknown mechanisms of homeostasis regulation.

### 6.6. Light Pollution, Skin Light-Receptions, and Modern Diseases

A large amount of evidence indicates that light is the most powerful environmental cue that entrains circadian clocks. Neuropsin (OPN5) expressed in murine skin melanocytes has been demonstrated to mediate local light-dependent induction of the circadian clock and photoentrainment of circadian clocks in murine skin [145]. This challenges the long-held dogma that peripheral circadian clocks in mammals are exclusively synchronized by the central circadian pacemaker in the SCN via ocular light perception and shows the presence of a direct light response system for circadian entrainment in murine skin, similar to pathways previously described in nonvertebrate species and certain non-mammalian vertebrates. Moreover, an interesting study was reported formerly in which investigators used light pulse stimuli to the popliteal region of volunteers, and several surprising findings were noted, including that the magnitude and direction of the phase shift of the circadian clock showed a systematic relationship with the timing of the light pulse, indicating that circadian photoentrainment can also be mediated through an extraocular route in humans [153]. The challenge ahead is to determine which skin cell type serves as the primary system for sensing and calculating light and eventually translating it into circadian entrainment responses. One of the most pronounced candidates is melanocytes, which can sense and respond to light through photosensitive opsins and can communicate by secreting various neuroendocrine factors and/or unknown bioactive substances.

The light-receiving sensory structures within the skin, considered to be remnants from evolution, have remained well preserved even after drastic variations in human lifestyles like sunscreen and clothing. Modern nighttime artificial light exposure has become a common phenomenon and leads to circadian misalignment, which produces symptoms that range from fairly mild, such as jet lag, to life-limiting conditions such as depression, metabolic disorders, cardiovascular or gastrointestinal disease, and oncogenesis [212]. On the other hand, timed bright light therapy benefits many sleep and circadian clock disorders caused by jet lag, shift-work, age-related insomnia, and advanced or delayed sleep phase syndrome [213,214]. Despite recent advances, much is still unknown about how our skin “sees” light. Further studies are required to clarify more details about light reception and signal transmission in the skin, not only to illustrate how life on earth interacts with light in the evolutionary process of harnessing light energy but also to unravel the underlying mechanisms behind modern diseases that are related to widespread light pollution and its consequent circadian clock disruptions.

## 7. Conclusions

**Figure 2:**
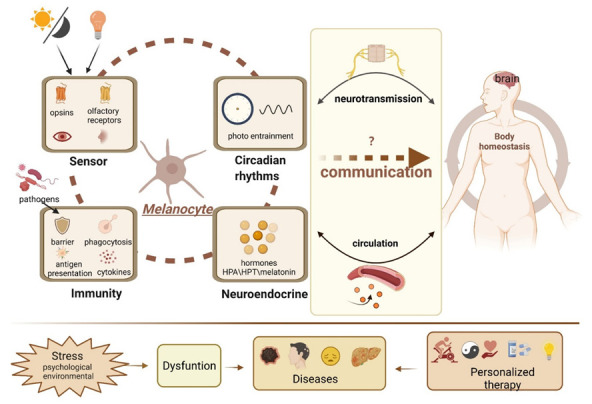
The Non-classical Function of Melanocytes and Their Role in Systemic Homeostasis. The skin melanocytes are empowered with sensory and computing capabilities and have a complex functional network, including ectopic light perception, neuroendocrine interaction, immune defense, and peripheral circadian rhythm to counteract environmental stressors and maintain local homeostasis. These complex functional systems maintain global homeostasis by communicating bidirectionally with the nervous and endocrine systems. Overloaded stressors, such as prolonged exposure to psychosocial stress and environmental stress, ultimately lead to imbalanced regulation, such as rhythm disturbances and endocrine disorders, and lead to the onset of disease. Thus, a more in-depth understanding of these relationships in future studies will pave a bright path for the management of melanoma, mood disorders, and metabolic disorders. The figure was created using BioRender.

The last several decades have seen tremendous advances in our understanding of the biology of melanocytes. We now adequately recognize that melanocytes are amazing cells and should not be solely considered as a “melanin factory.” They can carry out multiple functions, acting as epidermal photoreceptors and an active partner of the skin’s immune and neuroendocrine systems and responding quickly and effectively to environmental information to regulate the local and systemic homeostasis, as shown in Figure 2. However, critical questions as to why melanocytes are equipped with so many functional devices and to what extent these physiological functions can be exerted and even whether there is coordination between these functions, such as neuroendocrine activities and circadian rhythms, should be further integrated. Despite how these activities affect local physiology, how they transmit and amplify signals and even integrate with other remote tissues remains relatively unknown. Undoubtedly, the numerous activities of melanocytes are complex and highly controlled processes. Therefore, deciphering the mechanisms of the communication process can potentially contribute to gaining insight into the pleiotropic roles of melanocytes within the organism and can open a completely new horizon for revolutionizing the way diseases will be diagnosed and treated in the future.

## Figures and Tables

**Table 1:** Expression and Function of Opsins in Melanocytes.

Opsin	Species	Potential Effect	Reference
OPN1	Mouse	Not shown	[146]
	Human	Not shown	[127,129]
OPN2	Mouse	Mediate UVA-induced immediate pigment darkening	[126,135,147]
		Regulate clock genes and melanogenesis responding to white light	
	Human	Mediate UVR-induced early melanin synthesis	[127,128,129]
OPN3	Human	Sense blue light and regulate long-lasting hyperpigmentation	[129,130,131,132,133]
		Negatively regulate pigmentation through interaction with MC1R	
		Regulate the survival of melanocytes	
OPN4	Mouse	Mediate UVA-induced immediate pigment darkening	[146,147,148]
		Regulate clock genes and melanogenesis responding to white light	
		Mediate UVA-related proliferation and apoptosis	
		Mediate thermal activation of clock genes	
	Human	Photoreceptor of blue light	[124]
OPN5	Mouse	Local circadian photoentrainment	[145]
	Human	Regulate UVR-induced melanogenesis	[129,130]

**Table 2:** Locations and Functions of Extracutaneous Melanocytes.

Location	Function	Reference
Heart	Support the stiffness and mechanical properties of the cardiac valves	[172,173,174,175,176]
	Reduce ROS	
	Regulate electrical and structural remodel	
	Maintain the endolymphatic potential	
	Regulate cochlear development	
	Stabilize the intrastriatal fluid–blood barrier	
	Protect from noise and ototoxic	
Inner ear	Reduce ROS	[180,181,182,190,191,193]
Eye	Eye pigmentation and protection against UV	[189,194,198,199]
	Support the normal vasculature of the choroid	
	Induce chemokine secretion and monocyte Migration	
Sebaceous glands	May be a source of melanocyte stem cells	[200]
Brain	Neuroendocrine and detoxification	[195]
Adipose	Abate oxidative stress and inflammation	[196,197]

## References

[1] Lin J.Y., Fisher D.E. (2007). Melanocyte biology and skin pigmentation. Nature.

[2] Sulaimon S.S., Kitchell B.E. (2003). The biology of melanocytes. Vet. Dermatol..

[3] Cordero R.J.B., Casadevall A. (2020). Melanin. Curr. Biol..

[4] ElObeid A.S., Kamal-Eldin A., Abdelhalim M.A.K., Haseeb A.M. (2017). Pharmacological Properties of Melanin and its Function in Health. Basic Clin. Pharmacol. Toxicol..

[5] Plonka P.M., Passeron T., Brenner M., Tobin D.J., Shibahara S., Thomas A., Slominski A., Kadekaro A.L., Hershkovitz D., Peters E. (2009). What are melanocytes really doing all day long…?. Exp. Dermatol..

[6] Slominski A.T., Zmijewski M.A., Skobowiat C., Zbytek B., Slominski R.M., Steketee J.D. (2012). Sensing the environment: Regulation of local and global homeostasis by the skin’s neuroendocrine system. Adv. Anat. Embryol. Cell Biol..

[7] Slominski A., Paus R., Schadendorf D. (1993). Melanocytes as “sensory” and regulatory cells in the epidermis. J. Theor. Biol..

[8] Cichorek M., Wachulska M., Stasiewicz A., Tyminska A. (2013). Skin melanocytes: Biology and development. Postepy Dermatol. Alergol..

[9] Slominski A. (2009). Neuroendocrine activity of the melanocyte. Exp. Dermatol..

[10] Alexopoulos A., Chrousos G.P. (2016). Stress-related skin disorders. Rev. Endocr. Metab. Disord..

[11] Phan T.S., Schink L., Mann J., Merk V.M., Zwicky P., Mundt S., Simon D., Kulms D., Abraham S., Legler D.F. (2021). Keratinocytes control skin immune homeostasis through de novo-synthesized glucocorticoids. Sci. Adv..

[12] Paus R., Arck P. (2009). Neuroendocrine perspectives in alopecia areata: Does stress play a role?. J. Investig. Dermatol..

[13] Kotb El-Sayed M.I., Abd El-Ghany A.A., Mohamed R.R. (2018). Neural and Endocrinal Pathobiochemistry of Vitiligo: Comparative Study for a Hypothesized Mechanism. Front. Endocrinol..

[14] Zhang B., Ma S., Rachmin I., He M., Baral P., Choi S., Goncalves W.A., Shwartz Y., Fast E.M., Su Y. (2020). Hyperactivation of sympathetic nerves drives depletion of melanocyte stem cells. Nature.

[15] Bocheva G., Slominski R.M., Slominski A.T. (2019). Neuroendocrine Aspects of Skin Aging. Int. J. Mol. Sci..

[16] Theoharides T.C., Stewart J.M., Taracanova A., Conti P., Zouboulis C.C. (2016). Neuroendocrinology of the skin. Rev. Endocr. Metab. Disord..

[17] Leis K., Mazur E., Jablonska M.J., Kolan M., Galazka P. (2019). Endocrine systems of the skin. Postepy Dermatol. Alergol..

[18] Takeda K., Takahashi N.H., Shibahara S. (2007). Neuroendocrine functions of melanocytes: Beyond the skin-deep melanin maker. Tohoku J. Exp. Med..

[19] Rousseau K., Kauser S., Pritchard L.E., Warhurst A., Oliver R.L., Slominski A., Wei E.T., Thody A.J., Tobin D.J., White A. (2007). Proopiomelanocortin (POMC), the ACTH/melanocortin precursor, is secreted by human epidermal keratinocytes and melanocytes and stimulates melanogenesis. FASEB J..

[20] Spencer J.D., Schallreuter K.U. (2009). Regulation of pigmentation in human epidermal melanocytes by functional high-affinity beta-melanocyte-stimulating hormone/melanocortin-4 receptor signaling. Endocrinology.

[21] Slominski A. (1998). Identification of beta-endorphin, alpha-MSH and ACTH peptides in cultured human melanocytes, melanoma and squamous cell carcinoma cells by RP-HPLC. Exp. Dermatol..

[22] Bhm M., Metze D., Schulte U., Becher E., Luger T.A., Brzoska T. (2010). Detection of melanocortin-1 receptor antigenicity on human skin cells in culture and in situ. Exp. Dermatol..

[23] Farooqui J.Z., Medrano E.E., Abdel-Malek Z., Nordlund J. (1993). The expression of proopiomelanocortin and various POMC-derived peptides in mouse and human skin. Ann. N. Y. Acad. Sci..

[24] Slominski A., Ermak G., Hwang J., Chakraborty A., Mazurkiewicz J.E., Mihm M. (1995). Proopiomelanocortin, corticotropin releasing hormone and corticotropin releasing hormone receptor genes are expressed in human skin. FEBS Lett..

[25] Slominski A., Baker J., Ermak G., Chakraborty A., Pawelek J. (1996). Ultraviolet B stimulates production of corticotropin releasing factor (CRF) by human melanocytes. FEBS Lett..

[26] Kono M., Nagata H., Umemura S., Kawana S., Osamura R.Y. (2001). In situ expression of corticotropin-releasing hormone (CRH) and proopiomelanocortin (POMC) genes in human skin. FASEB J..

[27] Slominski A., Zbytek B., Szczesniewski A., Semak I., Kaminski J., Sweatman T., Wortsman J. (2005). CRH stimulation of corticosteroids production in melanocytes is mediated by ACTH. Am. J. Physiol. Endocrinol. Metab..

[28] Ramot Y., Bohm M., Paus R. (2021). Translational Neuroendocrinology of Human Skin: Concepts and Perspectives. Trends Mol. Med..

[29] Skobowiat C., Dowdy J.C., Sayre R.M., Tuckey R.C., Slominski A. (2011). Cutaneous hypothalamic-pituitary-adrenal axis homolog: Regulation by ultraviolet radiation. Am. J. Physiol. Endocrinol. Metab..

[30] Skobowiat C., Nejati R., Lu L., Williams R.W., Slominski A.T. (2013). Genetic variation of the cutaneous HPA axis: An analysis of UVB-induced differential responses. Gene.

[31] Slominski A.T., Slominski R.M., Raman C. (2021). UVB stimulates production of enkephalins and other neuropeptides by skin-resident cells. Proc. Natl. Acad. Sci. USA.

[32] Jozic I., Stojadinovic O., Kirsner R.S.F., Tomic-Canic M. (2015). Skin under the (Spot)-Light: Cross-Talk with the Central Hypothalamic-Pituitary-Adrenal (HPA) Axis. J. Investig. Dermatol..

[33] Sato H., Nagashima Y., Chrousos G.P., Ichihashi M., Funasak Y. (2002). The expression of corticotropin-releasing hormone in melanoma. Pigment. Cell Res..

[34] Kim M.H., Cho D., Kim H.J., Chong S.J., Lee K.H., Yu D.S., Park C.J., Lee J.Y., Cho B.K., Park H.J. (2006). Investigation of the corticotropin-releasing hormone-proopiomelanocortin axis in various skin tumours. Br. J. Dermatol..

[35] Eberle A.N., Rout B., Qi M.B., Bigliardi P.L. (2017). Synthetic Peptide Drugs for Targeting Skin Cancer: Malignant Melanoma and Melanotic Lesions. Curr. Med. Chem..

[36] Kingo K., Aunin E., Karelson M., Philips M.A., Ratsep R., Silm H., Vasar E., Soomets U., Koks S. (2007). Gene expression analysis of melanocortin system in vitiligo. J. Dermatol. Sci..

[37] Shaker O.G., Eltahlawi S.M., Tawfic S.O., Eltawdy A.M., Bedair N.I. (2016). Corticotropin-releasing hormone (CRH) and CRH receptor 1 gene expression in vitiligo. Clin. Exp. Dermatol..

[38] Spencer J., Gibbons N., Rokos H., Peters E., Wood J., Schallreuter K. (2007). Oxidative stress via hydrogen peroxide affects proopiomelanocortin peptides directly in the epidermis of patients with vitiligo. J. Investig. Dermatol..

[39] Mancino G., Miro C., Di Cicco E., Dentice M. (2021). Thyroid hormone action in epidermal development and homeostasis and its implications in the pathophysiology of the skin. J. Endocrinol. Investig..

[40] Bodo E., Kany B., Gaspar E., Knuver J., Kromminga A., Ramot Y., Biro T., Tiede S., van Beek N., Poeggeler B. (2010). Thyroid-stimulating hormone, a novel, locally produced modulator of human epidermal functions, is regulated by thyrotropin-releasing hormone and thyroid hormones. Endocrinology.

[41] Baldini E., Odorisio T., Sorrenti S., Catania A., Tartaglia F., Carbotta G., Pironi D., Rendina R., D’Armiento E., Persechino S. (2017). Vitiligo and Autoimmune Thyroid Disorders. Front. Endocrinol..

[42] Slominski A., Wortsman J., Kohn L., Ain K.B., Venkataraman G.M., Pisarchik A., Chung J.H., Giuliani C., Thornton M., Slugocki G. (2002). Expression of hypothalamic-pituitary-thyroid axis related genes in the human skin. J. Investig. Dermatol..

[43] Gaspar E., Nguyen-Thi K.T., Hardenbicker C., Tiede S., Plate C., Bodo E., Knuever J., Funk W., Biro T., Paus R. (2011). Thyrotropin-releasing hormone selectively stimulates human hair follicle pigmentation. J. Investig. Dermatol..

[44] Sandru F., Carsote M., Albu S.E., Dumitrascu M.C., Valea A. (2021). Vitiligo and chronic autoimmune thyroiditis. J. Med. Life.

[45] Liu M., Murphy E., Amerson E.H. (2016). Rethinking screening for thyroid autoimmunity in vitiligo. J. Am. Acad. Dermatol..

[46] Li D., Liang G., Calderone R., Bellanti J.A. (2019). Vitiligo and Hashimoto’s thyroiditis: Autoimmune diseases linked by clinical presentation, biochemical commonality, and autoimmune/oxidative stress-mediated toxicity pathogenesis. Med. Hypotheses.

[47] Ellerhorst J.A., Naderi A.A., Johnson M.K., Pelletier P., Prieto V.G., Diwan A.H., Johnson M.M., Gunn D.C., Yekell S., Grimm E.A. (2004). Expression of thyrotropin-releasing hormone by human melanoma and nevi. Clin. Cancer Res..

[48] Ellerhorst J.A., Sendi-Naderi A., Johnson M.K., Cooke C.P., Dang S.M., Diwan A.H. (2006). Human melanoma cells express functional receptors for thyroid-stimulating hormone. Endocr. Relat. Cancer.

[49] Ursu H. (2012). Functional TSH Receptors, Malignant Melanomas and Subclinical Hypothyroidism. Eur. Thyroid. J..

[50] Hou P., Liu D., Ji M., Liu Z., Engles J.M., Wahl R.L., Xing M. (2009). Induction of thyroid gene expression and radioiodine uptake in melanoma cells: Novel therapeutic implications. PLoS ONE.

[51] Scheau C., Draghici C., Ilie M.A., Lupu M., Caruntu C. (2021). Neuroendocrine Factors in Melanoma Pathogenesis. Cancers.

[52] Sevilla A., Chéret J., Slominski R.M., Slominski A.T., Paus R. (2022). Revisiting the role of melatonin in human melanocyte physiology: A skin context perspective. J. Pineal Res..

[53] Rusanova I., Martinez-Ruiz L., Florido J., Rodriguez-Santana C., Guerra-Librero A., Acuna-Castroviejo D., Escames G. (2019). Protective Effects of Melatonin on the Skin: Future Perspectives. Int. J. Mol. Sci..

[54] Kim T.K., Kleszczynski K., Janjetovic Z., Sweatman T., Lin Z., Li W., Reiter R.J., Fischer T.W., Slominski A.T. (2013). Metabolism of melatonin and biological activity of intermediates of melatoninergic pathway in human skin cells. FASEB J..

[55] Slominski A., Pisarchik A., Semak I., Sweatman T., Wortsman J., Szczesniewski A., Slugocki G., McNulty J., Kauser S., Tobin D.J. (2002). Serotoninergic and melatoninergic systems are fully expressed in human skin. FASEB J..

[56] Slominski A.T., Kim T.K., Kleszczynski K., Semak I., Janjetovic Z., Sweatman T., Skobowiat C., Steketee J.D., Lin Z., Postlethwaite A. (2020). Characterization of serotonin and N-acetylserotonin systems in the human epidermis and skin cells. J. Pineal Res..

[57] Slominski A., Pisarchik A., Johansson O., Jing C., Semak I., Slugocki G., Wortsman J. (2003). Tryptophan hydroxylase expression in human skin cells. Biochim. Biophys. Acta—Mol. Basis Dis..

[58] Kim T.K., Lin Z., Tidwell W.J., Li W., Slominski A.T. (2015). Melatonin and its metabolites accumulate in the human epidermis in vivo and inhibit proliferation and tyrosinase activity in epidermal melanocytes in vitro. Mol. Cell Endocrinol..

[59] Slominski A., Semak I., Pisarchik A., Sweatman T., Szczesniewski A., Wortsman J. (2002). Conversion ofL-tryptophan to serotonin and melatonin in human melanoma cells. FEBS Lett..

[60] Skobowiat C., Brozyna A.A., Janjetovic Z., Jeayeng S., Oak A.S.W., Kim T.K., Panich U., Reiter R.J., Slominski A.T. (2018). Melatonin and its derivatives counteract the ultraviolet B radiation-induced damage in human and porcine skin ex vivo. J. Pineal Res..

[61] Kleszczynski K., Kim T.K., Bilska B., Sarna M., Mokrzynski K., Stegemann A., Pyza E., Reiter R.J., Steinbrink K., Bohm M. (2019). Melatonin exerts oncostatic capacity and decreases melanogenesis in human MNT-1 melanoma cells. J. Pineal Res..

[62] Perdomo J., Quintana C., Gonzalez I., Hernandez I., Rubio S., Loro J.F., Reiter R.J., Estevez F., Quintana J. (2020). Melatonin Induces Melanogenesis in Human SK-MEL-1 Melanoma Cells Involving Glycogen Synthase Kinase-3 and Reactive Oxygen Species. Int. J. Mol. Sci..

[63] Janjetovic Z., Nahmias Z.P., Hanna S., Jarrett S.G., Kim T.K., Reiter R.J., Slominski A.T. (2014). Melatonin and its metabolites ameliorate ultraviolet B-induced damage in human epidermal keratinocytes. J. Pineal Res..

[64] Janjetovic Z., Jarrett S.G., Lee E.F., Duprey C., Reiter R.J., Slominski A.T. (2017). Melatonin and its metabolites protect human melanocytes against UVB-induced damage: Involvement of NRF2-mediated pathways. Sci Rep..

[65] Dong K., Goyarts E., Rella A., Pelle E., Wong Y.H., Pernodet N. (2020). Age Associated Decrease of MT-1 Melatonin Receptor in Human Dermal Skin Fibroblasts Impairs Protection Against UV-Induced DNA Damage. Int. J. Mol. Sci..

[66] Gillbro J.M., Marles L.K., Hibberts N.A., Schallreuter K.U. (2004). Autocrine catecholamine biosynthesis and the beta-adrenoceptor signal promote pigmentation in human epidermal melanocytes. J. Investig. Dermatol..

[67] Sivamani R.K., Porter S.M., Isseroff R.R. (2009). An epinephrine-dependent mechanism for the control of UV-induced pigmentation. J. Investig. Dermatol..

[68] Choi M.E., Yoo H., Lee H.R., Moon I.J., Lee W.J., Song Y., Chang S.E. (2020). Carvedilol, an Adrenergic Blocker, Suppresses Melanin Synthesis by Inhibiting the cAMP/CREB Signaling Pathway in Human Melanocytes and Ex Vivo Human Skin Culture. Int. J. Mol. Sci..

[69] Slominski A., Zmijewski M.A., Pawelek J. (2012). L-tyrosine and L-dihydroxyphenylalanine as hormone-like regulators of melanocyte functions. Pigment. Cell Melanoma Res..

[70] Ono K., Viet C.T., Ye Y., Dang D., Hitomi S., Toyono T., Inenaga K., Dolan J.C., Schmidt B.L. (2017). Cutaneous pigmentation modulates skin sensitivity via tyrosinase-dependent dopaminergic signalling. Sci. Rep..

[71] Mackintosh J.A. (2001). The antimicrobial properties of melanocytes, melanosomes and melanin and the evolution of black skin. J. Theor. Biol..

[72] Gasque P., Jaffar-Bandjee M.C. (2015). The immunology and inflammatory responses of human melanocytes in infectious diseases. J. Infect..

[73] Quaresma J. (2019). Organization of the Skin Immune System and Compartmentalized Immune Responses in Infectious Diseases. Clin. Microbiol. Rev..

[74] Harder J., Schroder J.M., Glaser R. (2013). The skin surface as antimicrobial barrier: Present concepts and future outlooks. Exp. Dermatol..

[75] Wang S., Liu D., Ning W., Xu A. (2015). Cytosolic dsDNA triggers apoptosis and pro-inflammatory cytokine production in normal human melanocytes. Exp. Dermatol..

[76] Iverson M.V. (2000). Hypothesis: Vitiligo virus. Pigment Cell Res..

[77] Erf G.F., Bersi T.K., Wang X., Sreekumar G.P., Smyth J.R. (2001). Herpesvirus connection in the expression of autoimmune vitiligo in Smyth line chickens. Pigment Cell Res..

[78] Kawamura T., Ogawa Y., Aoki R., Shimada S. (2014). Innate and intrinsic antiviral immunity in skin. J. Dermatol. Sci..

[79] Kabashima K., Honda T., Ginhoux F., Egawa G. (2019). The immunological anatomy of the skin. Nat. Rev. Immunol..

[80] Coates M., Blanchard S., MacLeod A.S. (2018). Innate antimicrobial immunity in the skin: A protective barrier against bacteria, viruses, and fungi. PLoS Pathog..

[81] Ahn J.H., Park T.J., Jin S.H., Kang H.Y. (2008). Human melanocytes express functional Toll-like receptor 4. Exp. Dermatol..

[82] Kawai T., Akira S. (2010). The role of pattern-recognition receptors in innate immunity: Update on Toll-like receptors. Nat. Immunol..

[83] Tapia C., Falconer M., Tempio F., Falcón F., López M., Fuentes M., Alburquenque C., Amaro J., Bucarey S., Di Nardo A. (2014). Melanocytes and melanin represent a first line of innate immunity against Candida albicans. Med. Mycol..

[84] Tam I., Dzierzega-Lecznar A., Stepien K. (2019). Differential expression of inflammatory cytokines and chemokines in lipopolysaccharide-stimulated melanocytes from lightly and darkly pigmented skin. Exp. Dermatol..

[85] Tam I., Stępień K. (2011). Secretion of proinflammatory cytokines by normal human melanocytes in response to lipopolysaccharide. Acta Biochim. Pol..

[86] Song H., Lee S., Choi G., Shin J. (2018). Repeated ultraviolet irradiation induces the expression of Toll-like receptor 4, IL-6, and IL-10 in neonatal human melanocytes. Photodermatol. Photoimmunol. Photomed..

[87] Koike S., Yamasaki K. (2020). Melanogenesis Connection with Innate Immunity and Toll-Like Receptors. Int. J. Mol. Sci..

[88] Vavricka C.J., Christensen B.M., Li J. (2010). Melanization in living organisms: A perspective of species evolution. Protein Cell.

[89] D’Alba L., Shawkey M.D. (2019). Melanosomes: Biogenesis, Properties, and Evolution of an Ancient Organelle. Physiol. Rev..

[90] Tolleson W.H. (2005). Human melanocyte biology, toxicology, and pathology. J. Environ. Sci. Health C Environ. Carcinog. Ecotoxicol. Rev..

[91] Montefiori D.C., Zhou J. (1991). Selective antiviral activity of synthetic soluble l-tyrosine and l-dopa melanins against human immunodeficiency virus in vitro. Antivir. Res..

[92] Ali S.M., Yosipovitch G. (2013). Skin pH: From basic science to basic skin care. Acta Derm. Venereol..

[93] Gunathilake R., Schurer N.Y., Shoo B.A., Celli A., Hachem J.P., Crumrine D., Sirimanna G., Feingold K.R., Mauro T.M., Elias P.M. (2009). pH-regulated mechanisms account for pigment-type differences in epidermal barrier function. J. Investig. Dermatol..

[94] Elias P.M., Menon G., Wetzel B.K., Williams J. (2010). Barrier requirements as the evolutionary “driver” of epidermal pigmentation in humans. Am. J. Hum. Biol..

[95] Lin T.K., Man M.Q., Abuabara K., Wakefield J.S., Sheu H.M., Tsai J.C., Lee C.H., Elias P.M. (2019). By protecting against cutaneous inflammation, epidermal pigmentation provided an additional advantage for ancestral humans. Evol. Appl..

[96] Jablonski N.G., Chaplin G. (2010). Colloquium paper: Human skin pigmentation as an adaptation to UV radiation. Proc. Natl. Acad. Sci. USA.

[97] Man M.Q., Lin T.K., Santiago J.L., Celli A., Zhong L., Huang Z.M., Roelandt T., Hupe M., Sundberg J.P., Silva K.A. (2014). Basis for enhanced barrier function of pigmented skin. J. Investig. Dermatol..

[98] Liu J., Man W.Y., Lv C.Z., Song S.P., Shi Y.J., Elias P.M., Man M.Q. (2010). Epidermal permeability barrier recovery is delayed in vitiligo-involved sites. Skin Pharmacol. Physiol..

[99] Koike S., Yamasaki K., Yamauchi T., Inoue M., Shimada-Ohmori R., Tsuchiyama K., Aiba S. (2018). Toll-like receptors 2 and 3 enhance melanogenesis and melanosome transport in human melanocytes. Pigment Cell Melanoma Res..

[100] Sun L., Pan S., Yang Y., Sun J., Liang D., Wang X., Xie X., Hu J. (2016). Toll-like receptor 9 regulates melanogenesis through NF-κB activation. Exp. Biol. Med..

[101] Le Poole I.C., van den Wijngaard R.M., Westerhof W., Verkruisen R.P., Dutrieux R.P., Dingemans K.P., Das P.K. (1993). Phagocytosis by normal human melanocytes in vitro. Exp. Cell Res..

[102] Smit N., Le Poole I., van den Wijngaard R., Tigges A., Westerhof W., Das P. (1993). Expression of different immunological markers by cultured human melanocytes. Arch. Dermatol. Res..

[103] Le Poole I., Mutis T., van den Wijngaard R., Westerhof W., Ottenhoff T., de Vries R., Das P. (1993). A novel, antigen-presenting function of melanocytes and its possible relationship to hypopigmentary disorders. J. Immunol..

[104] Lu Y., Zhu W.Y., Tan C., Yu G.H., Gu J.X. (2002). Melanocytes are potential immunocompetent cells: Evidence from recognition of immunological characteristics of cultured human melanocytes. Pigment Cell Res..

[105] Dalesio N.M., Barreto Ortiz S.F., Pluznick J.L., Berkowitz D.E. (2018). Olfactory, Taste, and Photo Sensory Receptors in Non-sensory Organs: It Just Makes Sense. Front. Physiol..

[106] Moraes M.N., de Assis L.V.M., Provencio I., Castrucci A.M.L. (2021). Opsins outside the eye and the skin: A more complex scenario than originally thought for a classical light sensor. Cell Tissue Res..

[107] Do M.T.H. (2019). Melanopsin and the Intrinsically Photosensitive Retinal Ganglion Cells: Biophysics to Behavior. Neuron.

[108] Lee S.J., Depoortere I., Hatt H. (2019). Therapeutic potential of ectopic olfactory and taste receptors. Nat. Rev. Drug Discov..

[109] Cheret J., Bertolini M., Ponce L., Lehmann J., Tsai T., Alam M., Hatt H., Paus R. (2018). Olfactory receptor OR2AT4 regulates human hair growth. Nat. Commun..

[110] Buscone S., Mardaryev A.N., Raafs B., Bikker J.W., Sticht C., Gretz N., Farjo N., Uzunbajakava N.E., Botchkareva N.V. (2017). A new path in defining light parameters for hair growth: Discovery and modulation of photoreceptors in human hair follicle. Lasers Surg. Med..

[111] Mignon C., Botchkareva N.V., Uzunbajakava N.E., Tobin D.J. (2016). Photobiomodulation devices for hair regrowth and wound healing: A therapy full of promise but a literature full of confusion. Exp. Dermatol..

[112] Castellano-Pellicena I., Uzunbajakava N.E., Mignon C., Raafs B., Botchkarev V.A., Thornton M.J. (2019). Does blue light restore human epidermal barrier function via activation of Opsin during cutaneous wound healing?. Lasers Surg. Med..

[113] Portillo M., Mataix M., Alonso-Juarranz M., Lorrio S., Villalba M., Rodriguez-Luna A., Gonzalez S. (2021). The Aqueous Extract of Polypodium leucotomos (Fernblock((R))) Regulates Opsin 3 and Prevents Photooxidation of Melanin Precursors on Skin Cells Exposed to Blue Light Emitted from Digital Devices. Antioxidants.

[114] Lan Y., Wang Y., Lu H. (2020). Opsin 3 is a key regulator of ultraviolet A-induced photoageing in human dermal fibroblast cells. Br. J. Dermatol..

[115] Lan Y., Zeng W., Dong X., Lu H. (2021). Opsin 5 is a key regulator of ultraviolet radiation-induced melanogenesis in human epidermal melanocytes. Br. J. Dermatol..

[116] Massberg D., Hatt H. (2018). Human Olfactory Receptors: Novel Cellular Functions Outside of the Nose. Physiol. Rev..

[117] Suh S., Choi E.H., Atanaskova Mesinkovska N. (2020). The expression of opsins in the human skin and its implications for photobiomodulation: A Systematic Review. Photodermatol. Photoimmunol. Photomed..

[118] Leung N.Y., Montell C. (2017). Unconventional Roles of Opsins. Annu. Rev. Cell Dev. Biol..

[119] Buhr E.D., Yue W.W., Ren X., Jiang Z., Liao H.W., Mei X., Vemaraju S., Nguyen M.T., Reed R.R., Lang R.A. (2015). Neuropsin (OPN5)-mediated photoentrainment of local circadian oscillators in mammalian retina and cornea. Proc. Natl. Acad. Sci. USA.

[120] Olinski L.E., Lin E.M., Oancea E. (2020). Illuminating insights into opsin 3 function in the skin. Adv. Biol. Regul..

[121] Provencio I., Jiang G., De Grip W.J., Hayes W.P., Rollag M.D. (1998). Melanopsin: An opsin in melanophores, brain, and eye. Proc. Natl. Acad. Sci. USA.

[122] Isoldi M.C., Rollag M.D., Castrucci A.M., Provencio I. (2005). Rhabdomeric phototransduction initiated by the vertebrate photopigment melanopsin. Proc. Natl. Acad. Sci. USA.

[123] Bertolesi G.E., McFarlane S. (2018). Seeing the light to change colour: An evolutionary perspective on the role of melanopsin in neuroendocrine circuits regulating light-mediated skin pigmentation. Pigment Cell Melanoma Res..

[124] Kusumoto J., Takeo M., Hashikawa K., Komori T., Tsuji T., Terashi H., Sakakibara S. (2020). OPN4 belongs to the photosensitive system of the human skin. Genes Cells.

[125] Miyashita Y., Moriya T., Kubota T., Yamada K., Asami K. (2001). Expression of opsin molecule in cultured murine melanocyte. J. Investig. Dermatol. Symp. Proc..

[126] Denda M., Fuziwara S. (2008). Visible radiation affects epidermal permeability barrier recovery: Selective effects of red and blue light. J. Investig. Dermatol..

[127] Tsutsumi M., Ikeyama K., Denda S., Nakanishi J., Fuziwara S., Aoki H., Denda M. (2009). Expressions of rod and cone photoreceptor-like proteins in human epidermis. Exp. Dermatol..

[128] Wicks N.L., Chan J.W., Najera J.A., Ciriello J.M., Oancea E. (2011). UVA phototransduction drives early melanin synthesis in human melanocytes. Curr. Biol..

[129] Haltaufderhyde K., Ozdeslik R.N., Wicks N.L., Najera J.A., Oancea E. (2015). Opsin expression in human epidermal skin. Photochem. Photobiol..

[130] Hu Q.M., Yi W.J., Su M.Y., Jiang S., Xu S.Z., Lei T.C. (2017). Induction of retinal-dependent calcium influx in human melanocytes by UVA or UVB radiation contributes to the stimulation of melanosome transfer. Cell Prolif..

[131] Regazzetti C., Sormani L., Debayle D., Bernerd F., Tulic M.K., De Donatis G.M., Chignon-Sicard B., Rocchi S., Passeron T. (2018). Melanocytes Sense Blue Light and Regulate Pigmentation through Opsin-3. J. Investig. Dermatol..

[132] Ozdeslik R.N., Olinski L.E., Trieu M.M., Oprian D.D., Oancea E. (2019). Human nonvisual opsin 3 regulates pigmentation of epidermal melanocytes through functional interaction with melanocortin 1 receptor. Proc. Natl. Acad. Sci. USA.

[133] Wang Y., Lan Y., Lu H. (2020). Opsin3 Downregulation Induces Apoptosis of Human Epidermal Melanocytes via Mitochondrial Pathway. Photochem. Photobiol..

[134] de Assis L.V.M., Moraes M.N., Magalhaes-Marques K.K., Castrucci A.M.L. (2018). Melanopsin and rhodopsin mediate UVA-induced immediate pigment darkening: Unravelling the photosensitive system of the skin. Eur. J. Cell Biol..

[135] Jin H., Zou Z., Chang H., Shen Q., Liu L., Xing D. (2021). Photobiomodulation therapy for hair regeneration: A synergetic activation of β-CATENIN in hair follicle stem cells by ROS and paracrine WNTs. Stem Cell Rep..

[136] Le Duff F., Fontas E., Guardoli D., Lacour J., Passeron T. (2022). HeaLED: Assessment of skin healing under light-emitting diode (LED) exposure-A randomized controlled study versus placebo. Lasers Surg. Med..

[137] Diogo M., Campos T., Fonseca E., Pavani C., Horliana A., Fernandes K., Bussadori S., Fantin F., Leite D., Yamamoto Â. (2021). Effect of Blue Light on Acne Vulgaris: A Systematic Review. Sensors.

[138] Spinella A., de Pinto M., Galluzzo C., Testoni S., Macripò P., Lumetti F., Parenti L., Magnani L., Sandri G., Bajocchi G. (2022). Photobiomodulation Therapy: A New Light in the Treatment of Systemic Sclerosis Skin Ulcers. Rheumatol. Ther..

[139] Kemény L., Varga E., Novak Z. (2019). Advances in phototherapy for psoriasis and atopic dermatitis. Expert Rev. Clin. Immunol..

[140] Allada R., Bass J. (2021). Circadian Mechanisms in Medicine. N. Engl. J. Med..

[141] Tanioka M., Yamada H., Doi M., Bando H., Yamaguchi Y., Nishigori C., Okamura H. (2009). Molecular clocks in mouse skin. J. Investig. Dermatol..

[142] Sandu C., Dumas M., Malan A., Sambakhe D., Marteau C., Nizard C., Schnebert S., Perrier E., Challet E., Pevet P. (2012). Human skin keratinocytes, melanocytes, and fibroblasts contain distinct circadian clock machineries. Cell Mol. Life Sci..

[143] Plikus M.V., Andersen B. (2018). Skin as a window to body-clock time. Proc. Natl. Acad. Sci. USA.

[144] Upton B.A., Diaz N.M., Gordon S.A., Van Gelder R.N., Buhr E.D., Lang R.A. (2021). Evolutionary Constraint on Visual and Nonvisual Mammalian Opsins. J. Biol. Rhythm..

[145] Buhr E.D., Vemaraju S., Diaz N., Lang R.A., Van Gelder R.N. (2019). Neuropsin (OPN5) Mediates Local Light-Dependent Induction of Circadian Clock Genes and Circadian Photoentrainment in Exposed Murine Skin. Curr. Biol..

[146] de Assis L.V., Moraes M.N., da Silveira Cruz-Machado S., Castrucci A.M. (2016). The effect of white light on normal and malignant murine melanocytes: A link between opsins, clock genes, and melanogenesis. Biochim. Biophys. Acta.

[147] de Assis L.V.M., Mendes D., Silva M.M., Kinker G.S., Pereira-Lima I., Moraes M.N., Menck C.F.M., Castrucci A.M.L. (2020). Melanopsin mediates UVA-dependent modulation of proliferation, pigmentation, apoptosis, and molecular clock in normal and malignant melanocytes. Biochim. Biophys. Acta Mol. Cell Res..

[148] Moraes M.N., de Assis L.V.M., Magalhaes-Marques K.K., Poletini M.O., de Lima L., Castrucci A.M.L. (2017). Melanopsin, a Canonical Light Receptor, Mediates Thermal Activation of Clock Genes. Sci. Rep..

[149] Solessio E., Engbretson G.A. (1993). Antagonistic chromatic mechanisms in photoreceptors of the parietal eye of lizards. Nature.

[150] Okano T., Yoshizawa T., Fukada Y. (1994). Pinopsin is a chicken pineal photoreceptive molecule. Nature.

[151] Zhao X., Haeseleer F., Fariss R.N., Huang J., Baehr W., Milam A.H., Palczewski K. (1997). Molecular cloning and localization of rhodopsin kinase in the mammalian pineal. Vis. Neurosci..

[152] Czeisler C.A., Shanahan T.L., Klerman E.B., Martens H., Brotman D.J., Emens J.S., Klein T., Rizzo J.F. (1995). Suppression of melatonin secretion in some blind patients by exposure to bright light. N. Engl. J. Med..

[153] Campbell S.S., Murphy P.J. (1998). Extraocular circadian phototransduction in humans. Science.

[154] Busse D., Kudella P., Gruning N.M., Gisselmann G., Stander S., Luger T., Jacobsen F., Steinstrasser L., Paus R., Gkogkolou P. (2014). A synthetic sandalwood odorant induces wound-healing processes in human keratinocytes via the olfactory receptor OR2AT4. J. Investig. Dermatol..

[155] Pavan B., Dalpiaz A. (2017). Odorants could elicit repair processes in melanized neuronal and skin cells. Neural Regen Res..

[156] Spehr M., Gisselmann G., Poplawski A., Riffell J.A., Wetzel C.H., Zimmer R.K., Hatt H. (2003). Identification of a testicular odorant receptor mediating human sperm chemotaxis. Science.

[157] Griffin C.A., Kafadar K.A., Pavlath G.K. (2009). MOR23 promotes muscle regeneration and regulates cell adhesion and migration. Dev. Cell.

[158] Weber L., Al-Refae K., Ebbert J., Jagers P., Altmuller J., Becker C., Hahn S., Gisselmann G., Hatt H. (2017). Activation of odorant receptor in colorectal cancer cells leads to inhibition of cell proliferation and apoptosis. PLoS ONE.

[159] Sondersorg A.C., Busse D., Kyereme J., Rothermel M., Neufang G., Gisselmann G., Hatt H., Conrad H. (2014). Chemosensory information processing between keratinocytes and trigeminal neurons. J. Biol. Chem..

[160] Tsai T., Veitinger S., Peek I., Busse D., Eckardt J., Vladimirova D., Jovancevic N., Wojcik S., Gisselmann G., Altmuller J. (2017). Two olfactory receptors-OR2A4/7 and OR51B5-differentially affect epidermal proliferation and differentiation. Exp. Dermatol..

[161] Gelis L., Jovancevic N., Veitinger S., Mandal B., Arndt H.D., Neuhaus E.M., Hatt H. (2016). Functional Characterization of the Odorant Receptor 51E2 in Human Melanocytes. J. Biol. Chem..

[162] Wojcik S., Weidinger D., Stander S., Luger T., Hatt H., Jovancevic N. (2018). Functional characterization of the extranasal OR2A4/7 expressed in human melanocytes. Exp. Dermatol..

[163] Adameyko I., Lallemend F. (2010). Glial versus melanocyte cell fate choice: Schwann cell precursors as a cellular origin of melanocytes. Cell Mol. Life Sci..

[164] Lerner M.R., Reagan J., Gyorgyi T., Roby A. (1988). Olfaction by melanophores: What does it mean?. Proc. Natl. Acad. Sci. USA.

[165] Karlsson J.O., Svensson S.P., Martensson L.G., Odman S., Elwing H., Lundstrom K.I. (1994). Effects of odorants on pigment aggregation and cAMP in fish melanophores. Pigment. Cell Res..

[166] Gelis L., Jovancevic N., Bechara F.G., Neuhaus E.M., Hatt H. (2017). Functional expression of olfactory receptors in human primary melanoma and melanoma metastasis. Exp. Dermatol..

[167] Ranzani M., Iyer V., Ibarra-Soria X., Del Castillo Velasco-Herrera M., Garnett M., Logan D., Adams D.J. (2017). Revisiting olfactory receptors as putative drivers of cancer. Wellcome Open Res..

[168] Suzuki T., Tomita Y. (2008). Recent advances in genetic analyses of oculocutaneous albinism types 2 and 4. J. Dermatol. Sci..

[169] Nichols S.E., Reams W.M. (1960). The occurrence and morphogenesis of melanocytes in the connective tissues of the PET/MCV mouse strain. J. Embryol. Exp. Morphol..

[170] Mjaatvedt C.H., Kern C.B., Norris R.A., Fairey S., Cave C.L. (2005). Normal distribution of melanocytes in the mouse heart. Anat. Rec. A Discov. Mol. Cell Evol. Biol..

[171] Brito F.C., Kos L. (2008). Timeline and distribution of melanocyte precursors in the mouse heart. Pigment. Cell Melanoma Res..

[172] Yajima I., Larue L. (2008). The location of heart melanocytes is specified and the level of pigmentation in the heart may correlate with coat color. Pigment. Cell Melanoma Res..

[173] Sanchez-Pina J., Lorenzale M., Fernandez M.C., Duran A.C., Sans-Coma V., Fernandez B. (2019). Pigmentation of the aortic and pulmonary valves in C57BL/6J x Balb/cByJ hybrid mice of different coat colours. Anat. Histol. Embryol..

[174] Balani K., Brito F.C., Kos L., Agarwal A. (2009). Melanocyte pigmentation stiffens murine cardiac tricuspid valve leaflet. J. R. Soc. Interface.

[175] Carneiro F., Kruithof B.P., Balani K., Agarwal A., Gaussin V., Kos L. (2015). Relationships between melanocytes, mechanical properties and extracellular matrix composition in mouse heart valves. J. Long Term Eff. Med. Implant..

[176] Levin M.D., Lu M.M., Petrenko N.B., Hawkins B.J., Gupta T.H., Lang D., Buckley P.T., Jochems J., Liu F., Spurney C.F. (2009). Melanocyte-like cells in the heart and pulmonary veins contribute to atrial arrhythmia triggers. J. Clin. Investig..

[177] Hwang H., Liu F., Levin M.D., Patel V.V. (2014). Isolating primary melanocyte-like cells from the mouse heart. J. Vis. Exp..

[178] Hwang H., Liu F., Petrenko N.B., Huang J., Schillinger K.J., Patel V.V. (2015). Cardiac melanocytes influence atrial reactive oxygen species involved with electrical and structural remodeling in mice. Physiol. Rep..

[179] Tsai W.C., Chan Y.H., Hsueh C.H., Everett T.H., Chang P.C., Choi E.K., Olaopa M.A., Lin S.F., Shen C., Kudela M.A. (2016). Small conductance calcium-activated potassium current and the mechanism of atrial arrhythmia in mice with dysfunctional melanocyte-like cells. Heart Rhythm..

[180] Tachibana M. (1999). Sound needs sound melanocytes to be heard. Pigment. Cell Res..

[181] Zhang W., Dai M., Fridberger A., Hassan A., Degagne J., Neng L., Zhang F., He W., Ren T., Trune D. (2012). Perivascular-resident macrophage-like melanocytes in the inner ear are essential for the integrity of the intrastrial fluid-blood barrier. Proc. Natl. Acad. Sci. USA.

[182] Zhang F., Dai M., Neng L., Zhang J.H., Zhi Z., Fridberger A., Shi X. (2013). Perivascular macrophage-like melanocyte responsiveness to acoustic trauma--a salient feature of strial barrier associated hearing loss. FASEB J..

[183] Huang S., Song J., He C., Cai X., Yuan K., Mei L., Feng Y. (2021). Genetic insights, disease mechanisms, and biological therapeutics for Waardenburg syndrome. Gene Ther..

[184] Andrade A., Pithon M. (2011). Alezzandrini syndrome: Report of a sixth clinical case. Dermatology.

[185] O’Keefe G.A., Rao N.A. (2017). Vogt-Koyanagi-Harada disease. Surv. Ophthalmol..

[186] Lee T.L., Lin P.H., Chen P.L., Hong J.B., Wu C.C. (2020). Hereditary Hearing Impairment with Cutaneous Abnormalities. Genes.

[187] Anbar T.S., El-Badry M.M., McGrath J.A., Abdel-Azim E.S. (2015). Most individuals with either segmental or non-segmental vitiligo display evidence of bilateral cochlear dysfunction. Br. J. Dermatol..

[188] Ertugrul G., Ertugrul S., Soylemez E. (2020). Investigation of hearing and outer hair cell function of the cochlea in patients with vitiligo. Dermatol. Ther..

[189] Genedy R., Assal S., Gomaa A., Almakkawy B., Elariny A. (2021). Ocular and auditory abnormalities in patients with vitiligo: A case-control study. Clin. Exp. Dermatol..

[190] Mujica-Mota M.A., Schermbrucker J., Daniel S.J. (2015). Eye color as a risk factor for acquired sensorineural hearing loss: A review. Hear. Res..

[191] Wrzesniok D., Beberok A., Otreba M., Buszman E. (2015). Gentamicin affects melanogenesis in normal human melanocytes. Cutan. Ocul. Toxicol..

[192] Wrzesniok D., Rok J., Beberok A., Rzepka Z., Respondek M., Pilawa B., Zdybel M., Delijewski M., Buszman E. (2019). Kanamycin induces free radicals formation in melanocytes: An important factor for aminoglycosides ototoxicity. J. Cell Biochem..

[193] Murillo-Cuesta S., Contreras J., Zurita E., Cediel R., Cantero M., Varela-Nieto I., Montoliu L. (2010). Melanin precursors prevent premature age-related and noise-induced hearing loss in albino mice. Pigment Cell Melanoma Res..

[194] Shibuya H., Watanabe R., Maeno A., Ichimura K., Tamura M., Wakana S., Shiroishi T., Ohba K., Takeda K., Tomita H. (2018). Melanocytes contribute to the vasculature of the choroid. Genes Genet. Syst..

[195] Gudjohnsen S., Atacho D., Gesbert F., Raposo G., Hurbain I., Larue L., Steingrimsson E., Petersen P. (2015). Meningeal Melanocytes in the Mouse: Distribution and Dependence on Mitf. Front. Neuroanat..

[196] Randhawa M., Huff T., Valencia J.C., Younossi Z., Chandhoke V., Hearing V.J., Baranova A. (2009). Evidence for the ectopic synthesis of melanin in human adipose tissue. FASEB J..

[197] Page S., Chandhoke V., Baranova A. (2011). Melanin and melanogenesis in adipose tissue: Possible mechanisms for abating oxidative stress and inflammation?. Obes. Rev..

[198] Jehs T., Faber C., Udsen M.S., Jager M.J., Clark S.J., Nissen M.H. (2016). Induction of Chemokine Secretion and Monocyte Migration by Human Choroidal Melanocytes in Response to Proinflammatory Cytokines. Investig. Ophthalmol. Vis. Sci..

[199] Nag T.C. (2015). Ultrastructural changes in the melanocytes of aging human choroid. Micron.

[200] Jang Y.H., Kim S.L., Lee J.S., Kwon K.Y., Lee S.J., Kim D.W., Lee W.J. (2014). Possible existence of melanocytes or melanoblasts in human sebaceous glands. Ann. Dermatol..

[201] Vandamme N., Berx G. (2019). From neural crest cells to melanocytes: Cellular plasticity during development and beyond. Cell Mol. Life Sci..

[202] Adameyko I., Lallemend F., Aquino J.B., Pereira J.A., Topilko P., Muller T., Fritz N., Beljajeva A., Mochii M., Liste I. (2009). Schwann cell precursors from nerve innervation are a cellular origin of melanocytes in skin. Cell.

[203] Tatarakis D., Cang Z., Wu X., Sharma P.P., Karikomi M., MacLean A.L., Nie Q., Schilling T.F. (2021). Single-cell transcriptomic analysis of zebrafish cranial neural crest reveals spatiotemporal regulation of lineage decisions during development. Cell Rep..

[204] Kaucka M., Szarowska B., Kavkova M., Kastriti M.E., Kameneva P., Schmidt I., Peskova L., Joven Araus A., Simon A., Kaiser J. (2021). Nerve-associated Schwann cell precursors contribute extracutaneous melanocytes to the heart, inner ear, supraorbital locations and brain meninges. Cell Mol. Life Sci..

[205] Bonnamour G., Soret R., Pilon N. (2021). Dhh-expressing Schwann cell precursors contribute to skin and cochlear melanocytes, but not to vestibular melanocytes. Pigment Cell Melanoma Res..

[206] Slominski A., Brożyna A., Tuckey R. (2017). Cutaneous Glucocorticoidogenesis and Cortisol Signaling Are Defective in Psoriasis. J. Investig. Dermatol..

[207] Paus R., Theoharides T., Arck P. (2006). Neuroimmunoendocrine circuitry of the ‘brain-skin connection’. Trends Immunol..

[208] Martins A., Ascenso A., Ribeiro H., Marto J. (2020). The Brain-Skin Connection and the Pathogenesis of Psoriasis: A Review with a Focus on the Serotonergic System. Cells.

[209] Slominski A. (2015). Ultraviolet radiation (UVR) activates central neuro-endocrine-immune system. Photodermatol. Photoimmunol. Photomed..

[210] Fell G., Robinson K., Mao J., Woolf C., Fisher D. (2014). Skin β-endorphin mediates addiction to UV light. Cell.

[211] Zhu H., Wang N., Yao L., Chen Q., Zhang R., Qian J., Hou Y., Guo W., Fan S., Liu S. (2018). Moderate UV Exposure Enhances Learning and Memory by Promoting a Novel Glutamate Biosynthetic Pathway in the Brain. Cell.

[212] Sancar A., Van Gelder R. (2021). Clocks, cancer, and chronochemotherapy. Science.

[213] Cuesta M., Boudreau P., Cermakian N., Boivin D. (2017). Rapid resetting of human peripheral clocks by phototherapy during simulated night shift work. Sci. Rep..

[214] Ruan W., Yuan X., Eltzschig H. (2021). Circadian rhythm as a therapeutic target. Nat. Rev. Drug Discov..

